# Framework for Quantifying the Efficiency of Competing Signal Transmission Modes in Proteins

**DOI:** 10.1021/jacs.5c14419

**Published:** 2026-01-07

**Authors:** Anil Kumar Sahoo, Hossein Batebi, Richard Schwarzl, Markus S. Miettinen, Roland R. Netz

**Affiliations:** † Fachbereich Physik, 9166 Freie Universität Berlin, Arnimallee 14, Berlin 14195, Germany; ‡ Max Planck Institute of Colloids and Interfaces, Am Mühlenberg 1, Potsdam 14476, Germany; § Computational Biology Unit, Department of Chemistry, University of Bergen, Bergen 5007, Norway; ∥ Centre for Condensed Matter Theory, Department of Physics, Indian Institute of Science, Bangalore 560012, India

## Abstract

On the microscopic level, biological signal transmission relies on coordinated transient structural changes in allosteric proteins that involve sensor and effector modules. The time scales and microscopic details of signal transmission in proteins are often unclear, despite a plethora of structural information on signaling proteins. Based on linear-response theory, we develop the theoretical framework to define frequency-dependent force and displacement transmit functions through proteins and, more generally, viscoelastic media. Transmit functions quantify the fraction of a local time-dependent perturbation at one site, be it a deformation, a force or a combination thereof, that survives at a distant site. They are defined in terms of equilibrium fluctuations from simulations or experimental observations. We apply the framework to our all-atom molecular dynamics simulations of a bacterial histidine kinase protein extensively studied in experiments. For the isolated coiled-coil (CC) motif that connects sensor and effector modules, our analysis reveals that signal propagation through the CC is possible via shift, splay, and twist deformation modes, which is confirmed by simulations of the entire protein. Based on mutation experiments, we infer that the most relevant mode for the biological function of the histidine kinase is the splay deformation. For the β_2_-adrenergic receptor, a transmembrane protein involved in the G-protein signaling pathway, we compare signal transmission across its different structural domains involved in receptor activation.

## Graphical abstract

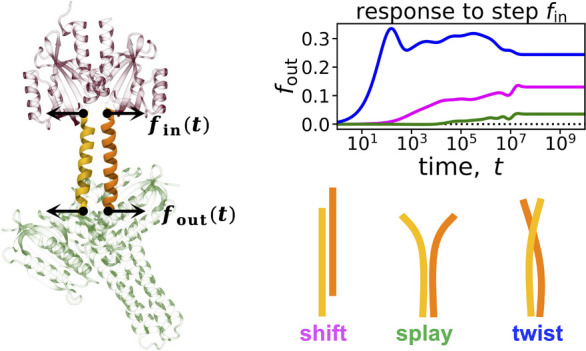

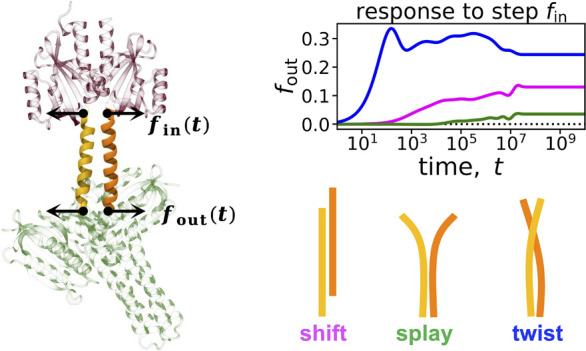

## Introduction

Signal transmission within a cell, between cells, and from the exterior to the interior of a cell is necessary for any form of life, be it bacteria, plants, or animals. Signal-transducing units, at the molecular level, involve signal receptors (sensors) that sense intracellular or environmental changes (such as photons, hormones, or pH) and corresponding effectors that are responsible for sparking a response.^1,2^ Sensor and effector modules are usually distinct domains of membrane-bound or cytosolic proteins.^3–5^ How information, at the molecular level, is transmitted through proteins has been the subject of intense research in the last few decades.^6–14^ Experimental techniques, such as NMR spectroscopy^15,16^ time-resolved crystallography^17,18^ cryo-electron microscopy,^19^ and single-molecule experiments^20–22^ have provided information about protein structure, dynamics, and mechanical signaling pathways. Computational approaches combining molecular simulation techniques^23–27^ and tools from information theory^28^ and graph theory^29–31^ along with various linear or nonlinear correlation analyses^32,33^ have provided molecular-level insights into protein allosteric communication pathways.^34,35^ A notable mention is the statistical mechanical approach to quantify the allosteric coupling between two distal regions in a protein via the thermodynamic coupling function.^36^ Many of the theoretical frameworks, however, use symmetric functions, which in principle are insufficient to quantify the directional, source-to-target transfer of bimolecular information^37^ and provide only stationary (time-independent) information about allosteric coupling. A combination of spectroscopy techniques and molecular dynamics (MD) simulations has led to recent insights into time-resolved processes of allosteric regulation.^38–43^ However, the quantitative relation between the dynamics of a protein and its signal transfer efficacy, which ultimately governs physiological response, is missing.

We show how to quantify the signal-transfer efficiency through proteins in terms of frequency-dependent force and displacement transmit functions. A force transmit function describes the fraction of the frequency-dependent force applied at the protein sensor position that survives at the effector position. The displacement transmit function is defined similarly but is based on spatial displacements. In fact, the presence of strongly correlated fluctuations at the sensor and effector positions, as quantified by two-point correlation functions, is not sufficient for efficient signal transmission, because equilibrium fluctuations produce a background that the signal has to compete with. Transmit functions weigh the correlation between the sensor and effector positions by the fluctuation magnitude and therefore quantify the efficiency of signal transmission. They are thus distinctly different from ordinary two-point correlation functions. Our theoretical framework builds on our previously developed convolution theory^44^ and is exact on the linear-response level.^45^ From the time-domain transmit function, the response of a protein to any temporal perturbation signal can be calculated by convolution, all one needs as input to our theory are time series of positions or displacements that can be obtained from MD simulations or single-molecule experiments, e.g., by fluorescence resonance energy transfer.^20^

We perform all-atom MD simulations of an engineered blue-light-regulated histidine kinase protein.^46^ In this synthetic enzyme, a coiled-coil (CC) motif connects the light-oxygen-voltage sensor module from *Bacillus subtilis* YtvA and the histidine kinase complex from the bacterium *Bradyrhizobium japonicum*, see Figure 1A. CCs are frequently found in various plant and bacterial signal transduction systems, that connect signal receptor and response or effector proteins.^4,47–53^ Signal transducers are commonly oligomeric domains and modular in architecture, produced by recombination of sensor and effector modules. Signaling through CCs leads to conformational rearrangements within effectors that trigger interaction with regulator proteins, starting signaling cascades. Studies investigating the role of CCs in signal propagation have, however, been largely limited to quantifying their stationary structural changes, such as helix pivoting or rotation^19,53^ in the presence of external stimuli. We study the dynamics of the isolated CC and the CC connected to the sensor and effector modules in terms of its shift, splay, and twist deformation modes. Computed transmit functions reveal that all modes are able to transmit signals. From the time-domain transmit functions, we derive transmit properties of different time-dependent signals. For a step signal, we find that transmission via the splay mode is markedly reduced by single-point mutations in the CC, whereas transmission via the twist mode is only minimally affected. Together with the experimental observation of light-induced splaying of the CC^17,54^ and experimental mutation studies,^5^ this suggests that splay is the most relevant deformation mode of the CC for the biological function of this sensor histidine kinase.^46^

**Figure 1:**
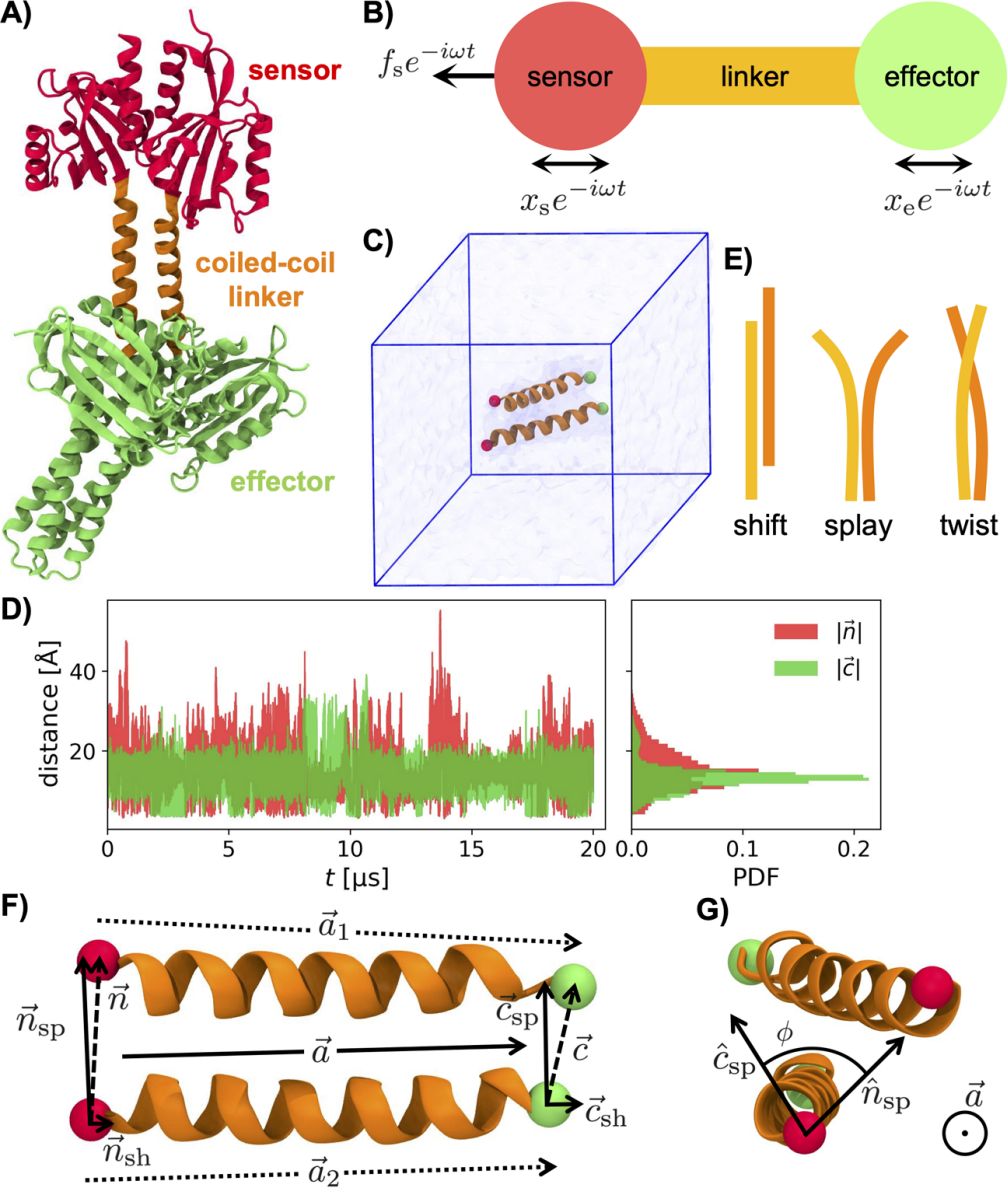
A) Picture of the full-length structure of the dark-adapted blue-light-regulated histidine kinase YF1 (PDB ID: 4GCZ),^63^ rendered using VMD.^64^ The sensor, linker, and effector modules of YF1 are homodimeric at the molecular level. (B) Schematic representation of signal transmission from the sensor to the effector site. (C) Simulation unit cell containing only the coiled-coil (CC) linker. Water and ions are not shown. (D) Time series and corresponding probability density functions (PDFs) for the N and C-termini distances of the isolated CC, |*n⃗*| and |*c⃗*|. (E) Schematic representation of shift, splay, and twist modes of the CC. (F) Schematics for defining the shift vectors *n⃗*_sh_, *c⃗*_sh_ and the splay vectors *n⃗*_sp_, *c⃗*_sp_ as the parallel and perpendicular components of the distance vectors *n⃗*, *c⃗* with respect to the symmetrized CC axis *a⃗* = (*a⃗*_1_ + *a⃗*_2_)/2. (G) Schematic for describing twist as rotation of splay unit vectors *n̂*_sp_ and *ĉ*_sp_ around the long-axis *a⃗*. The angle between *n̂*_sp_ and *ĉ*_sp_ is denoted as the twist angle ϕ.

To demonstrate the versatility of our method, we also apply our transmit-function formalism to a well-studied class A (rhodopsin-like) G-protein coupled receptor (GPCR), namely the β_2_-adrenergic receptor (β_2_-AR).^55–58^ GPCRs are the largest family of membrane proteins encoded in the human genome, responsible for detecting external signals and transducing them across the cell membrane via activation of intracellular G-proteins.^59–61^ These heptahelical transmembrane receptors are involved in virtually every physiological process and presently are targets for approximately 36% of all approved drugs in modern medicine.^62^ β_2_-AR regulates diverse physiological functions, including smooth muscle relaxation in airways and blood vessels, cardiac contractility, metabolic processes, and immune responses, and serves as a prototypical model for understanding GPCR activation, signaling pathways, and drug interactions. Our theoretical framework enables a quantitative assessment of signal propagation across β_2_-AR’s different structural domains that act as potential key allosteric hotspots during receptor activation.

## Results

### Theory of Transmit Functions

The input–output relation for a general responsive system can be quantified by two transmit functions: force and displacement transmission. Consider we apply to a system’s sensor (input) position a frequency-dependent force *F̃*_s_(ω) and to its effector (output) position a force *F̃*_e_(ω), see Figure 1B. In Fourier space, the sensor and effector positions *X̃*_s_(ω) and *X̃*_e_(ω) change to linear order as^44^

1 $$\begin{array}{ll} {\mathrm{X̃}}_{s}\left(\omega \right) & ={\mathrm{J̃}}_{\mathrm{self}}^{s}\left(\omega \right){\mathrm{F̃}}_{s}\left(\omega \right)+{\mathrm{J̃}}_{\mathrm{cross}}\left(\omega \right){\mathrm{F̃}}_{e}\left(\omega \right)\\ {\mathrm{X̃}}_{e}\left(\omega \right) & ={\mathrm{J̃}}_{\mathrm{self}}^{e}\left(\omega \right){\mathrm{F̃}}_{e}\left(\omega \right)+{\mathrm{J̃}}_{\mathrm{cross}}\left(\omega \right){\mathrm{F̃}}_{s}\left(\omega \right) \end{array}$$

where *J̃*_cross_(ω) and ${\mathrm{J̃}}_{\mathrm{self}}^{s/e}\left(\omega \right)$ are the frequency-dependent cross and self (sensor/effector side) linear response functions, respectively. Note that there is only a single cross-response, which is a consequence of Onsager’s celebrated reciprocal relation. However, the self-responses ${\mathrm{J̃}}_{\mathrm{self}}^{s}\left(\omega \right)$ and ${\mathrm{J̃}}_{\mathrm{self}}^{e}\left(\omega \right)$ are different for a general asymmetric system. It should be noted that eq 1 is exact on the linear response level and is applicable for any two positions in a protein. The force transmit function ${\mathrm{T̃}}_{F}^{s\to e}\left(\omega \right)$ is defined for the boundary condition of a stationary effector position *X̃*_e_ = 0, from eq 1 we obtain

2 $${\mathrm{T̃}}_{F}^{s\to e}\left(\omega \right)\equiv \frac{{\mathrm{F̃}}_{e}\left(\omega \right)}{-{\mathrm{F̃}}_{s}\left(\omega \right)}=\frac{{\mathrm{J̃}}_{\mathrm{cross}}\left(\omega \right)}{{\mathrm{J̃}}_{\mathrm{self}}^{e}\left(\omega \right)}$$

Similarly, setting *X̃*_s_ = 0 in eq 1, we obtain ${\mathrm{T̃}}_{F}^{e\to s}\equiv -{\mathrm{F̃}}_{s}/{\mathrm{F̃}}_{e}={\mathrm{J̃}}_{\mathrm{cross}}/{\mathrm{J̃}}_{\mathrm{self}}^{s}$ . Response functions are *causal*, i.e., there is no positional response before a force is applied, which implies *J̃*_cross_(ω) and *J̃*_self_(ω) have no poles in the upper half of the complex frequency plane. If furthermore *J̃*(ω) is nonzero in the upper half of the complex frequency plane, from eq 2 it follows that transmit functions are also causal.

The displacement transmit function ${\mathrm{T̃}}_{X}^{s\to e}\left(\omega \right)$ is defined as the ratio of the displacement of the effector site *X̃*_e_ divided by the displacement at the sensor site *X̃*_s_ under force-free boundary condition, *F̃*_e_ = 0. Inverting eq 1 yields

3 $$\begin{array}{ll} \left(\begin{array}{l} {\mathrm{F̃}}_{s}\\ {\mathrm{F̃}}_{e} \end{array}\right) & =\frac{1}{{\mathrm{J̃}}_{\mathrm{self}}^{s}{\mathrm{J̃}}_{\mathrm{self}}^{e}-{{\mathrm{J̃}}_{\mathrm{cross}}}^{2}}\left(\begin{array}{cc} {\mathrm{J̃}}_{\mathrm{self}}^{e} & -{\mathrm{J̃}}_{\mathrm{cross}}\\ -{\mathrm{J̃}}_{\mathrm{cross}} & {\mathrm{J̃}}_{\mathrm{self}}^{s} \end{array}\right)\left(\begin{array}{c} {\mathrm{X̃}}_{s}\\ {\mathrm{X̃}}_{e} \end{array}\right)\\ & =\left(\begin{array}{cc} {\mathrm{G̃}}_{\mathrm{self}}^{s} & {\mathrm{G̃}}_{\mathrm{cross}}\\ {\mathrm{G̃}}_{\mathrm{cross}} & {\mathrm{G̃}}_{\mathrm{self}}^{e} \end{array}\right)\left(\begin{array}{c} {\mathrm{X̃}}_{s}\\ {\mathrm{X̃}}_{e} \end{array}\right) \end{array}$$

where *G̃*’s are the moduli determined by inverting the response matrix $\left(\begin{array}{cc} {\mathrm{J̃}}_{\mathrm{self}}^{s} & {\mathrm{J̃}}_{\mathrm{cross}}\\ {\mathrm{J̃}}_{\mathrm{cross}} & {\mathrm{J̃}}_{\mathrm{self}}^{e} \end{array}\right)$ . From eq 3, we obtain

$${\mathrm{T̃}}_{X}^{s\to e}\equiv \frac{{\mathrm{X̃}}_{e}}{{\mathrm{X̃}}_{s}}=\frac{-{\mathrm{G̃}}_{\mathrm{cross}}}{{\mathrm{G̃}}_{\mathrm{self}}^{e}}=\frac{{\mathrm{J̃}}_{\mathrm{cross}}}{{\mathrm{J̃}}_{\mathrm{self}}^{s}}={\mathrm{T̃}}_{F}^{e\to s}$$ 4

Similarly, for *F̃*_s_ = 0, we obtain ${\mathrm{T̃}}_{X}^{e\to s}\equiv {\mathrm{X̃}}_{s}/{\mathrm{X̃}}_{e}={\mathrm{T̃}}_{F}^{s\to e}$ . Thus, force and inverse displacement transmit functions are the same. Note the striking resemblance between the transmit functions defined here and the transfer function which characterizes the output of a linear time-invariant system (e.g., an electric circuit consisting of resistors, inductors, and capacitors) in the context of signal processing.^65^

In practice, one need not apply external forces to determine linear response functions. *J*(*t*) can be obtained from equilibrium time-correlation functions, *C*(*t*), using the fluctuation–dissipation theorem^45^ (for a derivation, see Section S1 in the Supporting Information (SI))

5 $$J\left(t\right)=-\frac{1}{k_{B}T}\theta \left(t\right)\frac{d}{dt}C\left(t\right)$$

where *k*_B_ is the Boltzmann constant, *T* represents temperature, and θ­(*t*) is the Heaviside step function. The needed cross and self-correlation functions are defined as *C*_cross_(*t*) = ⟨*X*_s_(0)*X*_e_(*t*)⟩ and $C_{\mathrm{self}}^{s}\left(t\right)=\left\langle X_{s}\left(0\right)X_{s}\left(t\right)\right\rangle$ , $C_{\mathrm{self}}^{e}\left(t\right)=\left\langle X_{e}\left(0\right)X_{e}\left(t\right)\right\rangle$ , respectively (see Methods for details). Note that the positional trajectories of two sites within a protein, required for the calculation of *C*(*t*), can be obtained not only from MD simulations but also from single-molecule experiments.^20^

### Structural Stability of the CC Linker from MD Simulations

We consider the signaling protein histidine kinase introduced above to demonstrate the applicability of our theoretical framework to real systems. We perform explicit solvent all-atom MD simulations of the whole protein for the wild-type sequence (simulation details provided in Methods, and the simulation box shown in Figure S5 in the SI) and of the isolated CC linker for the wild-type and two different mutants. We mainly concentrate on the analysis of the CC-only simulations because of the superior data quality (20 μs-long simulation each) and validate our obtained results by comparison with the whole-protein simulation. For the isolated CC systems, a summary of simulation details is given in Methods, and the simulation unit cell is shown in Figure 1C. We find that the distribution of the N-termini distance at the sensor side is broader compared to the C-termini distance at the effector side, both distributions exhibit tails that reflect intermittent splaying of the α-helix termini (see Figure 1D,E). To check the long-time stability of secondary and tertiary structures of the isolated CC, we calculate three different order parameters: the fraction of native contacts *Q* between the two α-helices, the root-mean-square deviation (RMSD) of distances between the native and simulated CC structures (the former taken from the crystal structure of the full-length protein shown in Figure 1A), and the secondary structure (SS) content. These order parameters are defined in Section S2 in the SI, and their time-averaged values are provided in Figure S1. The overall configuration of the CC remains stable within 20 μs of simulation as indicated by an average RMSD of 2.5 Å. The two α-helices remain bound to each other (*Q* = 0.92) due to salt bridges (involving residues R135, E138, and E142) and hydrophobic interactions (involving residues L136, L139, L143, and V146).^5,63^ Individual α-helices also remain stable, as their fraction of SS content values exceed 0.85.

### The CC Linker Transmits Signals via Shift, Splay, and Twist Modes

We consider CC linker deformations that result from forces of equal magnitude and opposite direction acting on the two N-termini and the two C-termini. Thus, these deformations conserve linear momentum. We define three distinct deformation modes by different orientations of the terminal displacement vector with respect to the distance vector between the terminal groups: splay, where the displacement is parallel to the terminal separation, and shift and twist, where the displacement is perpendicular to the terminal separation (see schematics in Figure 1E). Splay conserves angular momentum, whereas shift and twist do not and are counteracted by a rotation of the sensor module. Since the typical rotational diffusion time of the sensor module, estimated to be of the order of 1 μs (see Methods), is much longer than shift and twist relaxation times, as will be shown below, shift and twist modes are nevertheless possible signal transmission modes.

The three signal transmission modes are obtained as follows. The position vectors of the N-termini and C-termini are *N⃗*_i_ and *C⃗*_i_, respectively, where *i* = 1, 2 refers to the first and second α-helix. These vectors are used to construct the separation vectors *c⃗* = *C⃗*_2_ – *C⃗*_1_, *n⃗* = *N⃗*_2_ – *N⃗*_1_, and *a⃗*_i_ = *C⃗*_i_ – *N⃗*_i_ (Figure 1F). From the end-to-end vectors *a⃗*_i_, we define the long axis of the CC as *a⃗* = (*a⃗*_1_ + *a⃗*_2_)/2. With respect to *a⃗*, we separate *n⃗* into the parallel component, shift (*n*_sh_ = |*n⃗*_sh_| = |*n⃗* · *â*|), and the perpendicular component, splay (*n*_sp_ = |*n⃗*_sp_| = |*n⃗* – *n⃗*_sh_|). Similarly, for the C-termini we obtain *c*_sh_ and *c*_sp_. The twist angle ϕ, depicted in Figure 1G, is obtained from the scalar product of the two splay unit vectors *n̂*_sp_ and *ĉ*_sp_ as ϕ = cos^–1^(*n̂*_sp_ · *ĉ*_sp_). We show time series of these deformation modes and the corresponding probability distribution functions in Figure S2 in the SI. Importantly, we find a strong bias toward clockwise rotation of the CC around its long axis *a⃗*, from the handedness plot obtained from the simulation (see Figure S2 in the SI), in agreement with the experimental observation of the left-handed supercoiling of the CC.^17^

The N and C-termini of the CC correspond to the sensor (s) and effector (e) side, respectively. For the shift mode, the two self-correlation functions are defined as $C_{\mathrm{self}}^{\mathrm{sh},s}=\left\langle n_{\mathrm{sh}}\left(0\right)n_{\mathrm{sh}}\left(t\right)\right\rangle$ and $C_{\mathrm{self}}^{\mathrm{sh},e}=\left\langle c_{\mathrm{sh}}\left(0\right)c_{\mathrm{sh}}\left(t\right)\right\rangle$ and the cross-correlation function is defined as $C_{\mathrm{cross}}^{\mathrm{sh}}=\left\langle n_{\mathrm{sh}}\left(0\right)c_{\mathrm{sh}}\left(t\right)\right\rangle =\left\langle c_{\mathrm{sh}}\left(0\right)n_{\mathrm{sh}}\left(t\right)\right\rangle$ . Correlation functions for the splay mode are defined by interchanging the shift quantities with the related splay quantities, e.g., $C_{\mathrm{self}}^{\mathrm{sp},s}=\left\langle n_{\mathrm{sp}}\left(0\right)n_{\mathrm{sp}}\left(t\right)\right\rangle$ . For the twist mode, the cross and two self-correlation functions are defined by the scalar product of the splay unit vectors as $C_{\mathrm{cross}}^{\mathrm{tw}}=\left\langle {\mathrm{n̂}}_{\mathrm{sp}}\left(0\right)\cdot {ĉ}_{\mathrm{sp}}\left(t\right)\right\rangle =\left\langle {ĉ}_{\mathrm{sp}}\left(0\right)\cdot {\mathrm{n̂}}_{\mathrm{sp}}\left(t\right)\right\rangle$ and $C_{\mathrm{self}}^{\mathrm{tw},s}=\left\langle {\mathrm{n̂}}_{\mathrm{sp}}\left(0\right)\cdot {\mathrm{n̂}}_{\mathrm{sp}}\left(t\right)\right\rangle$ and $C_{\mathrm{self}}^{\mathrm{tw},e}=\left\langle {ĉ}_{\mathrm{sp}}\left(0\right)\cdot {ĉ}_{\mathrm{sp}}\left(t\right)\right\rangle$ , respectively. To disentangle twist from overall CC rotation, we calculate twist correlation functions in the molecular coordinate frame obtained by removing the CC center-of-mass translation and rigid-body rotation around its principal axes at each time step.

We present results for the twist mode of the isolated CC in Figure 2 and for shift and splay modes in Figure S3 in the SI. Self-and cross-correlation functions are shown in Figure 2A, the corresponding response functions *J*(*t*), obtained using eq 5, are shown in Figure 2B. All self-and cross-response functions smoothly decay to zero. The relaxation time, defined as the largest decay time τ_max_ of a multiexponential fit of *J* (Section S4 in the SI), is found to be the fastest for twist (τ_max_ = 2.6 ns), followed by shift (τ_max_ = 7.1 ns) and splay (τ_max_ = 9.4 ns). As expected, for each mode, the two self-response functions are greater than the cross response at all times. The real and imaginary parts of the Fourier-transformed response functions, Re *J̃*(ω) and Im *J̃*(ω), are shown in Figure 2C,D. There is a distinct low-frequency plateau/peak in the real/imaginary part of the twist response. To obtain analytical representations, we fit multi-Debye functions (dashed lines) to the Fourier-transformed response functions *J̃*(ω) in Figure 2C,D (details are provided in Sections S3 and S4 in the SI). Inverse Fourier transforms of the fit functions (dashed lines) reproduce the simulated time-domain response functions (solid lines), *J*(*t*), in Figure 2B very well.

**Figure 2:**
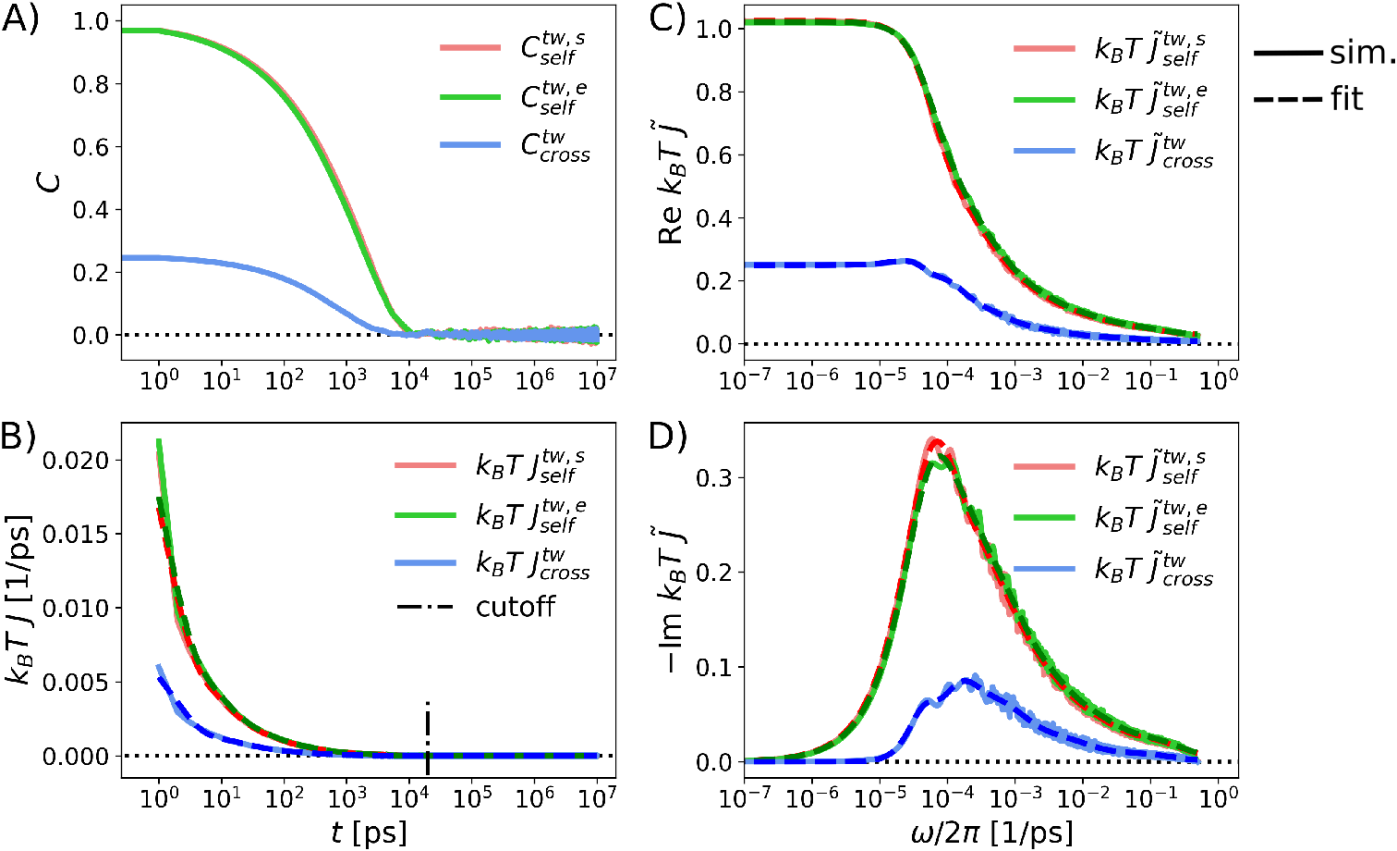
(A) Self- and cross-correlation functions (*C*_self_ and *C*_cross_) for the twist mode of the isolated CC. (B) Response functions, *J*, obtained using eq 5 from numerical derivatives of the simulated correlation functions in panel A are shown as solid lines. The vertical dash-dotted line represents the cutoff beyond which the response functions are set to zero to prevent noise artifacts when calculating Fourier transforms. (C) Real and (D) imaginary parts of *J̃*(ω) obtained from the discrete Fourier transform of *J*(*t*) are shown as solid lines. Dashed lines represent simultaneous fits to the real and imaginary parts by a sum of 10 Debye relaxation functions (for details, see Section S4 in the SI). Dashed lines in panel B represent the inverse Fourier transform of the fits in C and D.

Force transmit functions, *T̃*_F_(ω), obtained from the fitted *J̃*_self_(ω) and *J̃*_cross_(ω) using eq 2 are shown for all three different signaling modes in Figure 3. *T̃*_F_(ω) quantifies the system’s response to all possible excitation frequencies. We find that for the entire frequency range, force transmission through the twist mode (blue) is the highest, followed by the shift mode (red) and the splay mode (green). The differences are caused by energetic as well as dissipative effects, the latter include internal friction as well as hydrodynamic friction with the embedding solvent. From Re *T̃*_F_(ω) in Figure 3, it is also evident that no force transmission is possible via the shift and splay mode for an input signal of frequency >0.4 THz (ps^–1^) and >0.01 THz, respectively. These cutoff frequencies are similar to the water Debye mode at a frequency of about 0.02 THz,^66^ which suggests that the damping is partially due to the coupling to the hydration water. Except for the twist mode, the transmit functions are generally asymmetric, i.e., the sensor-to-effector side transmit function ${\mathrm{T̃}}_{F}^{s\to e}\left(\omega \right)$ and the effector-to-sensor side transmit function ${\mathrm{T̃}}_{F}^{e\to s}\left(\omega \right)$ are different. It should be noted that *T̃*_F_(ω) presented in Figure 3 characterizes the transmit properties of an isolated CC. The effects of added sensor and effector protein modules are obtained from the whole histidine kinase protein simulation (for details, see Section S6 and Figures S5–S7 in the SI). Although the absolute values of *T̃*_F_(ω) obtained from the simulation of the whole protein are larger than those of the isolated CC, the trends for the different signaling modes remain the same. We conclude that the reduced CC terminal fluctuations in the full construct in fact increase the signal transmission efficiency compared to the isolated CC scenario.

**Figure 3:**
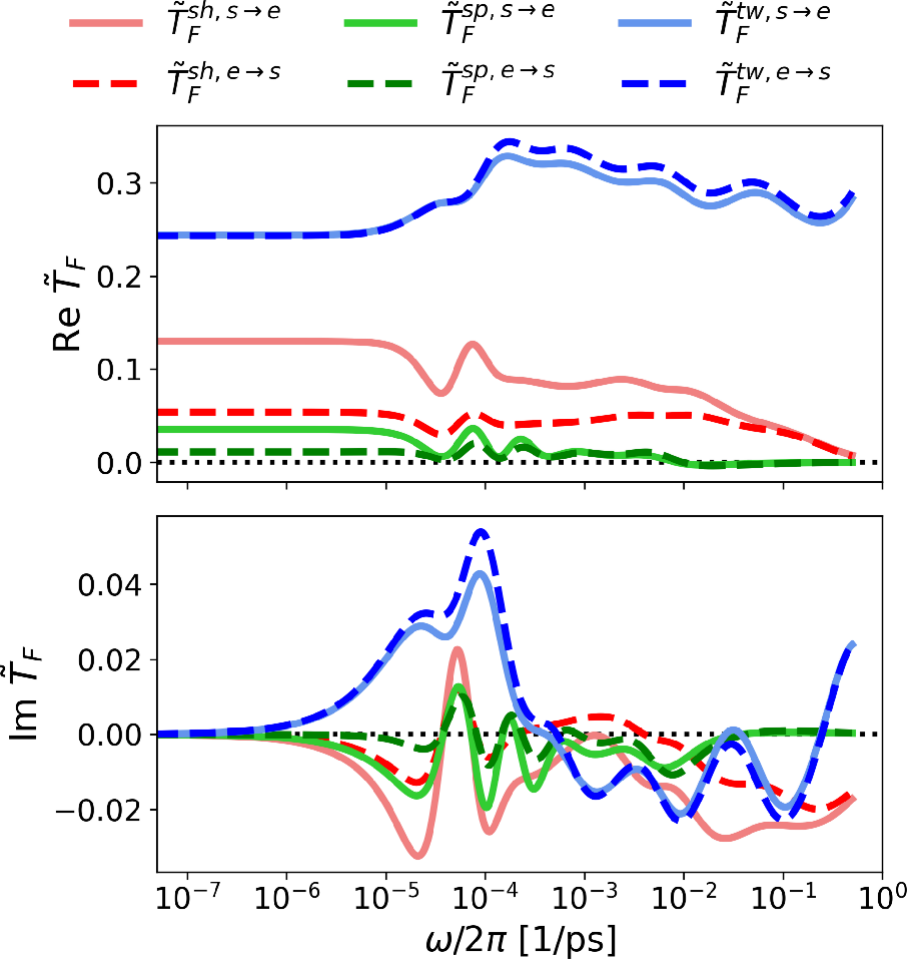
(Top) Real and (bottom) imaginary part of force transmit functions *T̃*_F_ for the shift (sh), splay (sp), and twist (tw) modes of the isolated CC obtained using analytical representations for the self- and cross-response functions (see details in Section S4 in the SI) according to eq 2. The sensor-to-effector (s → e) and effector-to-sensor (e → s) transmit functions are shown as solid and dashed lines, respectively.

Different force-field parameters can significantly alter protein stability and dynamics in simulations. We check this by performing simulations of the isolated CC using Amber99SB-ILDN^67,68^ and DES-Amber^69–71^ in addition to CHARMM36m^72^ used for the results presented in the main text. We find the signal transmission efficiency to be rather robust with respect to force-field variations, see Section S7 in the SI for details.

### Coupling between Deformation Modes of the CC

Berntsson et al.^17^ have experimentally observed superhelical coiling of the CC subsequent to its light-induced splaying apart of the histidine kinase sensor domains, which suggests coupling between splay and twist deformations.^53^ We quantify the coupling between different deformation modes by calculating the Pearson correlation coefficients (see Methods) using time-series data from the simulation of the whole histidine kinase protein, as summarized in Table 1. We observe that the shift-splay coupling is the highest, followed by the splay-twist coupling, regardless of whether considering data for the same ends (sensor and effector) or the different ends (cross). The shift-twist coupling is found to be insignificant in all cases. We indeed find nonvanishing splay-twist coupling for the sensor side, which explains the experimentally observed twisting by coupling to splay.

**Table 1:** Correlation Coefficients between Different Deformation Modes for the Same Ends (Sensor and Effector) and for Different Ends (Cross) of the CC from the Simulation of the Whole Histidine Kinase Protein^a^

Type	sh-sp	sp-tw	sh-tw
sensor	0.19 ± 0.06	0.10 ± 0.05	0.03 ± 0.09
effector	0.60 ± 0.05	-0.05 ± 0.12	0.03 ± 0.08
cross	0.31 ± 0.06	0.09 ± 0.10	0.05 ± 0.05

a Reported standard errors are estimated by block averaging each time series into three segments.

### Signal Transmission in the Time Domain

For practical purposes, signal transmission is conveniently characterized in the time domain. The force transmit function in the time domain, $T_{F}^{s\to e}\left(t\right)$ , is given by the inverse Fourier transform of ${\mathrm{T̃}}_{F}^{s\to e}\left(\omega \right)$ and describes the transmission of a δ-function input force signal *F*_s_(*t*) at the sensor side. Once $T_{F}^{s\to e}\left(t\right)$ is known, the transmitted force *F*_e_(*t*) at the effector side due to an arbitrary input force signal *F*_s_(*t*) can be obtained via convolution,

6 $$F_{e}\left(t\right)=\int_{0}^{\infty }T_{F}^{s\to e}\left(\tau \right)F_{s}\left(t-\tau \right)d\tau$$

*T*_F_(*t*) for the different signaling modes is shown in Figure 4A, and the method to obtain analytical representations for *T*_F_(*t*) from *T̃*_F_(ω) is explained in Section S5 in the SI. We find that the transmitted force signals for all the different modes decay rather quickly over a few picoseconds, which reflects the dominant fast time scale of the relaxation spectrum (see Tables S2–S4 in the SI).

**Figure 4:**
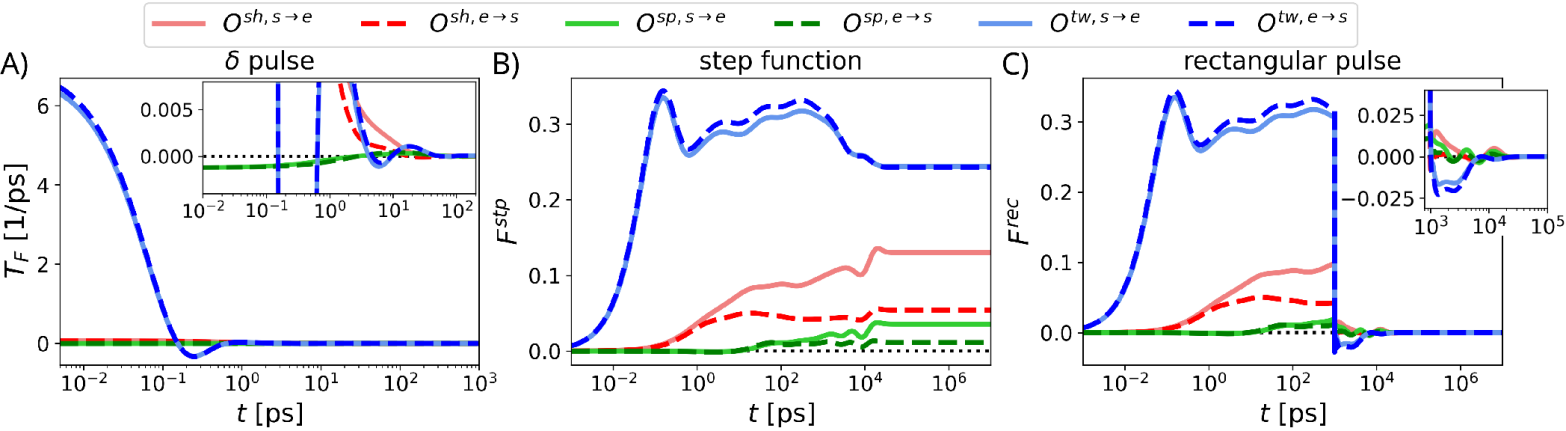
Transmitted force profiles for the different modes of the isolated CC for different input force signals: (A) δ pulse, (B) step function, and (C) rectangular pulse of width τ = 1 ns. Insets in panels A and C represent zoomed-in force profiles. The force profiles in B and C are rescaled by the input force strength.

In reality, signals are not transmitted via infinitely short δ-pulses but rather by pulses of finite duration. How efficiently the CC linker transmits such such a finite-duration input force signal depends on how quickly it responds to a suddenly imposed force and how quickly it relaxes back to its equilibrium state after the removal of force. To understand these complex dynamics, we discuss the transmitted output forces for force-step and rectangular-force-pulse input signals, $F_{e}^{\mathrm{stp}}\left(t\right)$ and $F_{e}^{\mathrm{rec}}\left(t\right)$ , both obtained from the convolution integral eq 6 (for details, see Section S5 in the SI). The step-response function is relevant for understanding activation of signaling proteins, since an agonist binding leads to a finite, sustained increase in the volume of the binding pocket, which can be modeled as a step-like increase in the separation between any two protein residues near the binding pocket. For a force switched on at *t* = 0, $F_{e}^{\mathrm{stp}}\left(t\right)$ is shown in Figure 4B for the different modes. The transmitted force rises steeply on the subpicosecond scale but stationary plateau values are reached for the different modes after 30–50 ns, which reflects the longer time scales of the relaxation spectrum (see Tables S2–S4 in the SI) and is around 2 orders of magnitude faster than the reported experimental time scale of 2 μs associated with light-induced conformational transitions within the sensor module.^17^ Signal transmission through all three modes is thus sufficiently rapid to avoid becoming rate-limiting. The plateau value of *F*_e_^stp^ is the highest for the twist mode, followed by shift and splay modes. In Figure 4C, we show the transmitted force $F_{e}^{\mathrm{rec}}\left(t\right)$ for a rectangular force pulse signal of duration τ = 1 ns that is switched on at *t* = 0. The transmitted force through each mode decays to zero rather quickly, after the removal of the applied force. Results for different durations of the rectangular force pulse of τ = 10^–2^, 10^–1^, 10^1^, 10^2^ ns are presented in Figure S4 in the SI. Note that due to the diffusive nature of the relevant conformational transitions, which can involve rotations of entire domains, signal propagation in larger proteins can imply substantially longer time scales than found from our analysis of the CC; signal propagation time scales in general depend on the length scale and complexity of the signal-transducing system.

### Robustness with Respect to Mutations in the CC

It has been experimentally demonstrated that single-point mutations within the CC reduce the signal response of the blue-light-regulated histidine kinase YF1.^5,63^ To investigate this using our framework, we perform MD simulations of two different experimentally studied mutants, Q133L and R135L. We find that these single-point mutations do not affect the overall coiled-coil conformation, as observed from their different structural order parameter values compared with that of the wild-type CC, provided in Figure S1 in the SI. However, the dynamics of these two mutants are completely different from each other and from the wild-type CC, as becomes clear from their step-force responses, $F_{e}^{\mathrm{stp}}\left(t\right)$ , shown in Figure 5.

**Figure 5:**
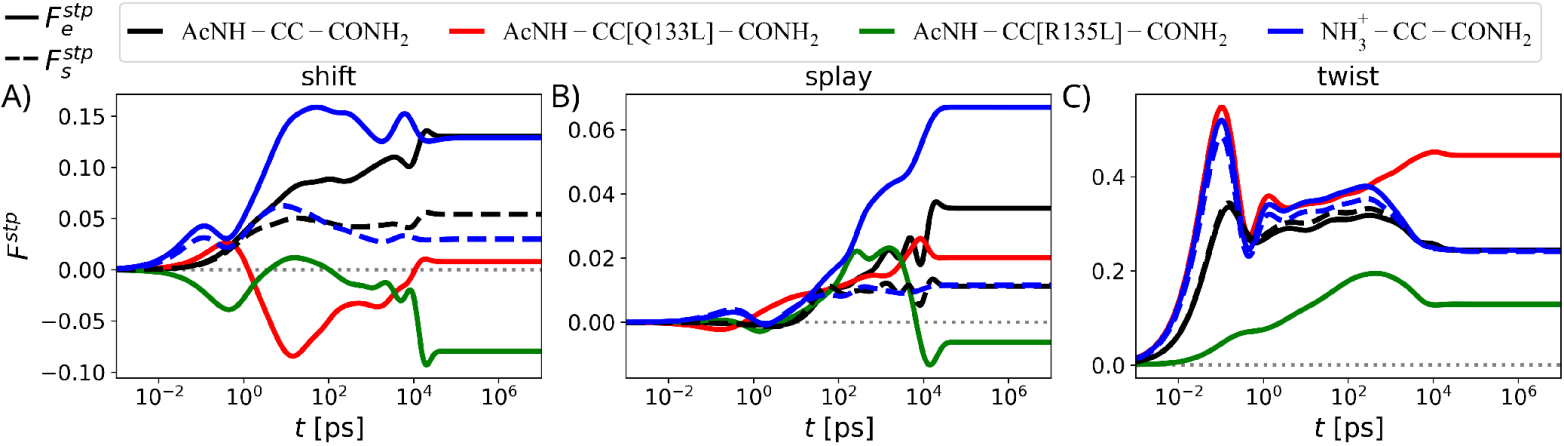
Effects of single-point mutations, Q133L (red), R135L (green), and charged ${\mathrm{NH}}_{3}^{+}$ N-termini (blue) on the step force transmit function, *F* ^stp^(*t*), of the isolated CC via (A) shift, (B) splay, and (C) twist modes. For comparison, *F* ^stp^ for each deformation mode is shown from Figure 4B for the wild-type CC with charge-neutral termini, denoted as AcNH–CC–CONH_2_ (black). Solid lines and broken lines (only shown for the wild-type CC with charge-neutral and charged N-temini) represent step responses at the effector side, $F_{e}^{\mathrm{stp}}\left(t\right)$ , and at the sensor side, $F_{s}^{\mathrm{stp}}\left(t\right)$ , respectively. All force profiles are rescaled by the input force strength.

For the mutant Q133L (red), the plateau values of $F_{e}^{\mathrm{stp}}\left(t\right)$ for the shift and splay modes are 16.7 and 1.8 times smaller, respectively, than that of the wild-type CC (black), as shown in Figure 5A,B. In contrast, the plateau value of $F_{e}^{\mathrm{stp}}\left(t\right)$ for the twist mode is 1.8 times larger than that of the wild-type CC (Figure 5C). Based on the absence of signaling for Q133L in experiments,^5^ the results in Figure 5 suggest that the signaling in the histidine kinase is not connected to the twist mode. For the mutant R135L (green), the shift and splay mode $F_{e}^{\mathrm{stp}}\left(t\right)$ plateau values are finite but negative. Compared to the wild-type CC, the $F_{e}^{\mathrm{stp}}\left(t\right)$ plateau value magnitudes for the shift, splay, and twist modes are reduced by 1.6, 5.7, and 1.9 times, respectively. In experiments, the signaling activity of the mutant R135L is reduced but not absent.^5^ In addition, structural characterizations by electron paramagnetic resonance spectroscopy and X-ray solution scattering have revealed that light induces a splaying apart of the sensor domains of the histidine kinase YF1 and hence of the N-termini of the wild-type CC.^17,54^ By integrating these experimental findings with our comparative analysis of mode-dependent step responses shown in Figure 5 for the wild-type and two mutated CC variants—which exhibit absent or reduced signaling—we conclude that the biologically relevant signaling mode in the histidine kinase is predominantly of the splay type.

Interestingly, our simulations reveal the twist mode to be the most stable mode with respect to the two single-point mutations (Figure 5C). Thus, it is conceivable that the CC linker might in a different biological context also function as a twist transmitter.

### Asymmetric Signal Transmission through the CC

Asymmetry could be important for the efficient information transfer from the sensor to the effector side. To look into this, we introduce the *rectification factor γ* as the ratio of sensor-to-effector (s → e) and effector-to-sensor (e → s) step-force transmission profile plateau values,

$$\gamma =\underset{t\to \infty }{\lim}\frac{F_{e}^{\mathrm{stp}}\left(t\right)}{F_{s}^{\mathrm{stp}}\left(t\right)}$$

From the results for the wild-type isolated CC shown in Figure 4B, we conclude that rectification for the splay mode is the highest, γ^sp^ = 3.2, followed by the shift mode, γ^sh^ = 2.4. In contrast, no rectification is observed for the twist mode, i.e., γ^tw^ = 1. These results can be rationalized by noting that the rectification factor is essentially determined by the ratio of the real parts of the low-frequency self-responses for the sensor and the effector ends (Figure S3C in the SI). To study the relation between the rectification factor and the structural asymmetry in more detail, we introduce an additional asymmetry between the sensor and effector ends of the CC by uncapping the sensor side α-helix termini, which thereby become positively charged at neutral pH, resulting in the structure ${\mathrm{NH}}_{3}^{+}$ –CC–CONH_2_ (note that the results presented in Figures 1–4 are obtained for the CC linker with charge-neutral end groups: AcNH–CC–CONH_2_). Though the step-force transmission profiles for AcNH–CC–CONH_2_ and ${\mathrm{NH}}_{3}^{+}$ –CC–CONH_2_ are qualitatively the same, we observe a pronounced difference in the plateau values of $F_{e}^{\mathrm{stp}}$ and $F_{s}^{\mathrm{stp}}$ for the shift and splay modes of ${\mathrm{NH}}_{3}^{+}$ –CC–CONH_2_ (see Figure 5A,B). However, the $F_{e}^{\mathrm{stp}}$ and $F_{s}^{\mathrm{stp}}$ plateau values for the twist mode are the same and remain unaffected in comparison to that of the charge neutral-termini system, AcNH–CC–CONH_2_ (Figure 5C). We thus find that the rectification factor γ can be tuned by changing chemical structures of sensor and effector-side terminal groups.

For the whole histidine kinase protein, which exhibits an additional asymmetry due to the added sensor and effector modules, we find rather similar rectification factor for the splay mode, γ^sp^ = 3.63, in comparison to the isolated wild-type CC. The corresponding value for twist (γ^tw^ = 0.82) is slightly reduced, while that for the shift (γ^sh^ = 0.67) is significantly reduced, compared to the isolated wild-type CC. Thus, the rectification factor for the splay mode is significantly larger than that for the shift and twist modes for the whole protein as well. Interestingly, this may be closely associated with the light-induced splaying of the CC linker observed in experiments.^17,54^ This observation suggests a possible correlation between the γ values of the different modes and their respective functional relevance.

### Signal Transmission in the GPCR β_2_-AR Protein

We have, so far, discussed signal propagation in the histidine kinase YF1 from the sensor to effector module through the CC linker, in which the two α-helices primarily exhibit shifting, splaying, and twisting. Cytosolic or transmembrane proteins, in general, show more complex deformations including melting and unfolding of helices. Our transmit function formalism is also applicable to the study of such cell signaling proteins with complex topologies, such as GPCRs having seven helical transmembrane domains.^59–61^ We consider a prototypical GPCR, the β_2_-AR protein (Figure 6A,B), which plays a crucial role in regulating cardiovascular, pulmonary, and metabolic functions. As a key target for bronchodilators in the treatment of asthma and chronic obstructive pulmonary disease,^73^ β_2_-AR is also implicated in biased signaling, where different ligands selectively activate distinct intracellular pathways, offering therapeutic potential beyond traditional agonists.^74^ Structural insights into β_2_-AR have significantly advanced our understanding of GPCR activation, ligand specificity, and allosteric modulation.^55^ Recent computational and cryo-electron microscopy studies have provided insights into the activation and deactivation mechanisms of β_2_-AR.^56–58,75^

**Figure 6:**
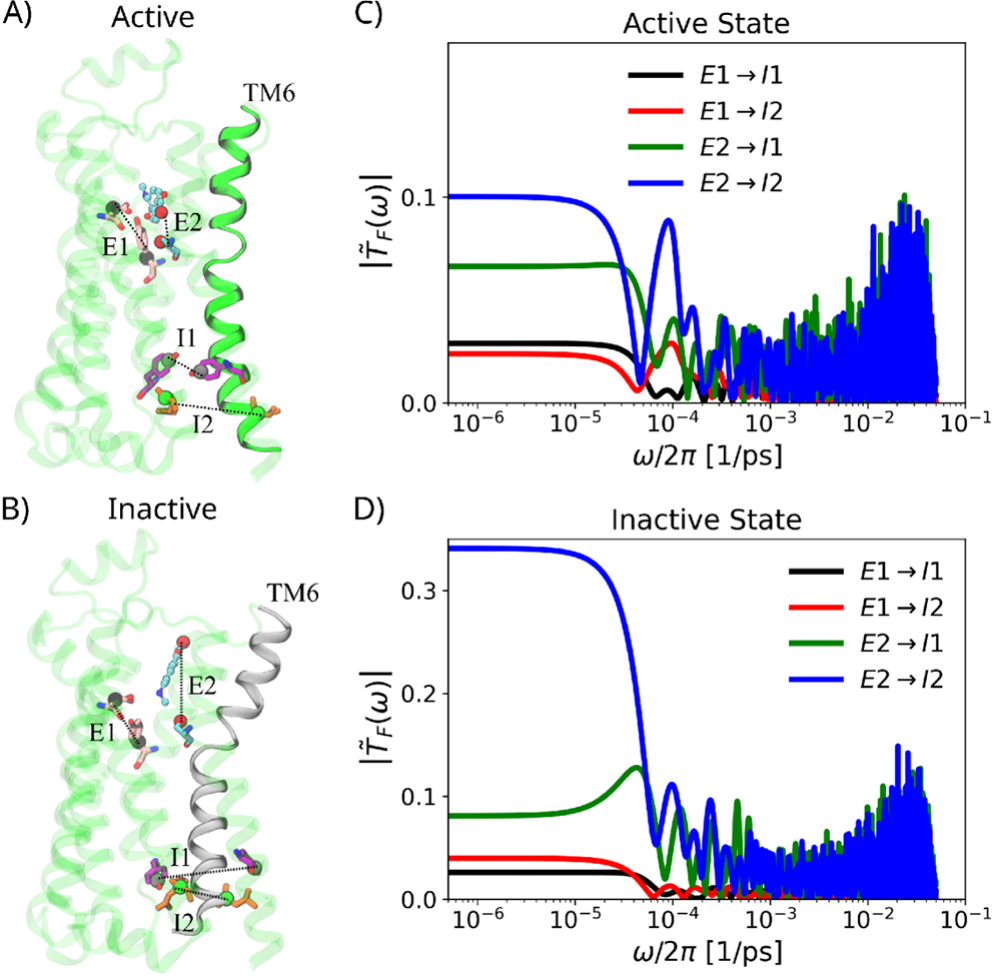
Signal transfer in the GPCR β_2_-adrenergic receptor (β_2_-AR) protein. A) Active and B) inactive state structures of β_2_-AR, for the whole simulation box see Figure S9A,B in the SI. The intracellular part of the transmembrane helix 6 (TM6) remains in the open or close conformation in the active or inactive state, respectively. Signal transmission is characterized by monitoring the two extracellular separations—E1 (distance between center-of-mass of residues D113 and Y316) and E2 (distance between the S207 side-chain oxygen and one oxygen of the agonist’s catechol group)—and the two intracellular separations—I1 (distance between center-of-mass of the side chains of Y219 and Y326) and I2 (distance between the backbone C_α_ atoms of R131 and L272). Modulus of the force transmit functions, |*T̃*_F_(ω)|, for signaling between different extracellular and intracellular parts of β_2_-AR in the C) active and D) inactive state.

We analyze two different simulation trajectories (see Methods) of a high-affinity agonist-bound β_2_-AR protein, embedded in a lipid membrane, in the (i) active and (ii) inactive state.^57,58^ The β_2_-AR transitions from its active to inactive state following decoupling of the G protein, accompanied by structural rearrangements that stabilize the inactive conformation.^58^ To evaluate whether our transmit function formalism can pinpoint critical hotspots of β_2_-AR’s allosteric signaling pathways, we focus on reaction coordinates known to play important roles in the receptor activation process.^56–58^ Those are the two extracellular separations, E1 and E2, and the two intracellular separations, I1 and I2, as depicted in Figure 6A,B. The selection of the reference atoms has been guided by functional considerations in addition to structural stability, as described below. E1 (D113–Y316 distance) and E2 (S207–agonist distance) monitor conformational changes in the ligand binding site. As a matter of fact, S207 is a key residue in the orthosteric binding pocket of β_2_-AR, where it forms direct hydrogen bonds with catechol ligands such as epinephrine, and plays a central role in receptor activation.^56^ I1 (Y219–Y326) is an important contact pair since reorientation of the side chains is functionally relevant and provides a meaningful indicator of gate opening on the intracellular side, in addition, Y326 is conserved in 99% of class A GPCRs. I2 (R131–L272 distance) captures the outward movement of transmembrane helix 6 (TM6) and the opening of the intracellular gate. Specifically, R131 belongs to the highly conserved DRY motif that stabilizes the inactive state and regulates conformational changes during activation.^76,77^ L272, located in TM6, has been deliberately selected as a reference point because the outward movement of TM6 at the cytoplasmic site is the hallmark of GPCR activation and is critical for G-protein coupling.^78^

Regardless of the different protein conformational ensembles sampled in the (i) active and (ii) inactive states, our transmit-function analysis shows that signaling from E2 to I2 is the most efficient, as is clear form the low-frequency plateau values of the modulus of the force transmit functions |*T̃*_F_(ω)| shown in Figure 6C,D. The efficient signaling between E2 (S207–agonist) and I2 (R131–L272) is consistent with a well-supported scenario based on experimental findings, according to which ligand engagement at S207 initiates conformational changes that propagate through the DRY motif to the cytoplasmic opening, where TM6 displacement exposes the intracellular space for G-protein binding.^56,76–78^ We thus demonstrate that our transmit-function formalism allows us to compare the efficiency of different signaling pathways in complex systems and to find optimal pairs of sensor and effector sites.

## Discussion and Conclusions

We present the theoretical framework to quantify the signal transmission between two distinct sites of a protein through different deformation modes, expressed in terms of the corresponding self- and cross-response functions. According to the fluctuation–dissipation theorem, the response functions are related to equilibrium time-correlation functions, which can be obtained from MD simulations as well as from single-molecule experiments. Note that in experiments, trajectories of separation coordinates typically include effects due to the coupling to measurement devices, which can be removed by using dynamic deconvolution theory.^44,79^ The displacement transmit function relates the correlations between two sites and the fluctuations at the site at which the input signal is applied, it thus quantifies the ratio of the output to the input signal and thus conveys more useful information than the often considered dynamic cross-correlation.

Applying our theoretical framework to the histidine kinase protein YF1, we demonstrate that all three deformation modes—twist, shift, and splay—in principle enable signal transfer from the sensor to the effector end of the CC. The experimentally observed splaying followed by superhelical coiling of the CC upon activation^17^ is expected due to the coupling between splay and twist deformation modes, as quantified by our analysis. Analysis of our simulation data for the wild-type CC and two single-point mutants^63^ suggests that splay is actually the signaling mode realized in the experimentally studied histidine kinase.^17,54^ Although twist, in principle, is a more effective mode of signal transmission, it does not conserve angular momentum and would therefore induce rotation of the sensor domain, this is probably why nature is not using it, at least for this protein construct. Previous experiments have indicated that the length of the CC linker, not only the actual linker sequence, is instrumental in determining the response to light signals.^80^ Our framework could in the future also be used to study the CC length-dependent signaling. In this paper, we have focused on signaling between identical deformation modes at the CC termini: twist-to-twist, splay-to-splay, and shift-to-shift. Off-diagonal signaling modes, that means coupling of e.g., splay at the sensor side to shift at the effector side, could be experimentally relevant and will be considered in future work.

For the GPCR β_2_-AR protein, our transmit-function formalism enables quantitative characterization of signal transmission across its structural domains, pinpointing critical allosteric sites implicated in activation. Our framework, with its capability to quantitatively map signal propagation between different domains, offers a versatile approach to study allosteric signal propagation in GPCRs, where complex topologies and conformational transitions challenge conventional analyses.^61^ In future work, it could be applied to quantify how ligand binding, G-protein coupling and decoupling, or recruitment of arrestin, which are GPCR-regulating proteins, modulate signal transmission across the receptor’s structural domains.

Signal transmission through general protein networks can be predicted from the response functions of individual components by repeated application of convolution relations for serial and parallel connections.^44,81^ Our study, thus, provides a way forward to relate atomic-level protein dynamics to large-scale intermolecular communications of biological signaling networks.

Our theory is formulated at the linear-response level and is therefore scale-invariant with respect to the input signal amplitude. To obtain the signal threshold beyond which the signal strength surpasses the noise background, one needs to compare the signal strength with the root-mean-square of the fluctuating force or displacement, similar to the definition of the signal-to-noise ratio in information theory.^82^

## Methods

### Models and Force-Field Parameters

#### Isolated CC Systems

From the crystal structure of the histidine kinase protein YF1 (PDB ID: 4GCZ), sensor (N-terminal) and effector (C-terminal) modules are deleted to obtain the structure of the isolated CC linker.^63^ The CC is composed of two parallel α-helices, each containing the same 23 residues ([126]­Ile-Thr-Glu-His-Gln-Gln-Thr-Gln-Ala-Arg-Leu-Gln-Glu-Leu-Gln-Ser-Glu-Leu-Val-His-Val-Ser-Arg[148]). The CC is simulated in a rhombic dodecahedron box of volume 227 nm^3^ filled with 7135 water molecules (and counterions needed to neutralize the system). CHARMM36m protein force field parameters,^72^ the TIP3P water model^83,84^ and ion parameters from ref.^85^ are used. Four different systems with changes of N-termini capping groups or a mutated residue are considered: ${\mathrm{NH}}_{3}^{+}$ –CC–CONH_2_, AcNH–CC–CONH_2_, AcNH–CC­[Q133L]–CONH_2_, AcNH–CC­[R135L]–CONH_2_. The acetyl (AcNH) group is used at the N-terminal and the “–CONH_2_” group is used at the C-terminal Arg, to simulate charge-neutral termini. The two mutated systems are selected from the study by Gleichmann *et al.* ^5^ For AcNH–CC–CONH_2_, two additional simulations using different protein force fields, Amber99SB-ILDN^67,68^ and DES-Amber^69–71^ are performed (for details, see Section S7 in the SI).

#### The Whole Histidine Kinase Protein

The histidine kinase YF1 (PDB ID: 4GCZ), with two flavin mononucleotide (FMN) cofactors bound to its sensor module and one adenosine diphosphate (ADP) bound to its effector module, is used as the initial structure. Missing hydrogen atoms are added using CHARMM-GUI.^86^ FMN is parametrized using the CHARMM General Force Field (CGenFF),^87^ while the protein and ADP are assigned parameters from the CHARMM36m force field.^72^ The complex is placed in a 15 × 15 × 15 nm^3^ cubic box and solvated with 110,941 TIP3P water molecules^83,84^ as shown in Figure S5 in the SI. The system is charge neutralized, and 0.10 M NaCl^85^ is added using the Monte Carlo ion placement method in CHARMM-GUI,^86^ ensuring a physiologically relevant ionic strength.

#### The GPCR β_2_-AR Protein

Details of the model building and equilibrium MD simulation protocol for the all-atom explicit solvent simulations of a high-affinity agonist, conformationally constrained epinephrine (c-Epi)-bound β_2_-AR protein, embedded into a pure 1-palmitoyl-2-oleyl-*sn*-glycero-3-phosphocholine (POPC) bilayer membrane, coupled to a GDP-bound G_s_ protein can be found in the recent publications by one of us.^57,58^ The CHARMM36m^72^ parameters for protein, the CHARMM36 ^88^ parameters for lipid, the TIP3P water model^83,84^ the parameters for c-Epi and GDP derived from CGenFF,^87^ and ion parameters from ref.^85^ are used. For our analysis, we consider from the above studies two different simulation trajectories of the agonist-bound β_2_-AR in the (i) active and (ii) inactive state, taken from simulations with the initial conformations denoted as structure 16 and structure 20, respectively, according to Papasergi-Scott et al.^58^ The simulation box for each of the two states is depicted in Figure S9 in the SI. Each simulation trajectory is of adequate duration (3.15 μs) with the data saved every 10 ps. For the simulation started with structure 20 that leads to the inactive state as monitored by the TM6 distance (I2) shown in Figure S9C in the SI, the trajectory excluding the first 200 ns, which corresponds to the transition from active-to-inactive state, is used for analysis.

### MD Simulation Details

For the whole histidine kinase protein and each isolated CC system, simulations are performed in the *NpT* ensemble at temperature *T* = 300 K and pressure *p* = 1 bar with periodic boundary conditions using the Gromacs package^89^ for 2 and 20 μs, respectively. The stochastic velocity rescaling thermostat^90^ with a time constant τ_*T*_ = 0.1 ps is used to control temperature, while for pressure control an isotropic Parrinello–Rahman barostat^91^ is used with a time constant τ_*p*_ = 2 ps and compressibility κ = 4.5 × 10^–5^ bar^–1^. The LINCS algorithm^92^ is used to constrain the bonds involving hydrogen atoms, allowing a time step Δ*t* = 2 fs. Electrostatic interactions are computed using the particle mesh Ewald method^93^ with a real-space cutoff distance of 1.2 nm, while van der Waals interactions are modeled using Lennard-Jones potentials with a cutoff distance of 1.2 nm where the resulting forces smoothly switch to zero between 1 to 1.2 nm. For data analysis, simulation trajectories are saved every 1 ps. Images are rendered using the visual molecular dynamics (VMD) software.^64^ Analysis is performed using in-house developed codes and Gromacs analysis modules.^89^ The time-correlation function for observables *A*(*t*) and *B*(*t*) is calculated as

$$\begin{array}{ll} C\left(\tau \right)=\frac{1}{L-\tau } & \int_{0}^{L-\tau }A\left(t\right)B\left(t+\tau \right)dt\\ & =\left\langle A\left(t\right)B\left(t+\tau \right)\right\rangle \end{array}$$

where τ is the time lag, and *L* is the trajectory length.

### Protein Rotational Relaxation Time

The rotational relaxation time τ_r_ of an object is related to the rotational diffusion coefficient as *D*_r_ = 1/2τ_r_ and is estimated from the Stokes’ rotational diffusion coefficient $D_{r}=k_{B}T/8\pi \eta R_{h}^{3}$ .^94^ Using the viscosity of the medium as that of water, η = 8.9 × 10^–1^ Pa·s, and the hydrodynamic radius as half of the largest length scale of the full-length protein YF1, *R*_h_ = 7 nm, we obtain τ_r_ = 2 μs, which is notably longer than the transmission relaxation times, by 1–2 orders of magnitude.

### Coupling between Deformation Modes

Pearson correlation coefficients between two different deformation modes, *i* and *j*, are obtained as

$$R_{ij}=\frac{\left\langle \left(x_{i}-\left\langle x_{i}\right\rangle \right)\times \left(x_{j}-\left\langle x_{j}\right\rangle \right)\right\rangle }{\sqrt{\left\langle {\left(x_{i}-\langle x_{i}\rangle \right)}^{2}\right\rangle }\sqrt{\left\langle {\left(x_{j}-\langle x_{j}\rangle \right)}^{2}\right\rangle }}$$

where ⟨·⟩ denotes the time average, and *x*_*i/j*_ refers to the time-series data for shift, splay, or twist at the same ends or at different ends of the CC. Using the time series data obtained from the simulation of the whole protein YF1, the computed correlation coefficients *R*_*ij*_ for *i* ≠ *j* (as *R*_*ii*_ = 1) are summarized in Table 1.

## Supplementary Material



## Data Availability

Simulation input files and analysis scripts are available online at GitHub repository, https://github.com/ComputBioSoftPhys/InfoTransfer_Proteins.

## Notes

Derivation of the fluctuation–dissipation theorem; definitions of structural order parameters, as well as their values; analytical expression of the force transmit function for a viscous bead–spring model; fitting procedure for response functions; procedure to obtain time-domain transmit functions, as well as the parameters; results for the full histidine kinase protein YF1; results on force field effects (PDF)
