# Supplementary material for: Framework for Quantifying the Efficiency of Competing Signal Transmission Modes in Proteins
Source: J Am Chem Soc. 2026 Jan 7;148(2):2283–94. doi: 10.1021/jacs.5c14419 (PMC12833803; doi:10.1021/jacs.5c14419)
Supplement: Supplementary file 1 [file ja5c14419_si_001.pdf]

Supporting Information: Framework for Quantifying the Efficiency of Competing Signal Transmission Modes in Proteins

Anil Kumar Sahoo,[†,‡] Hossein Batebi,[†] Richard Schwarzl,[†] Markus S. Miettinen,[†,¶] and Roland R. Netz[*,†,§]

†*Fachbereich Physik, Freie Universität Berlin, Arnimallee 14, 14195 Berlin, Germany*

‡*Max Planck Institute of Colloids and Interfaces, Am Mühlenberg 1, 14476 Potsdam, Germany*

¶*Computational Biology Unit, Department of Chemistry, University of Bergen, 5007 Bergen, Norway*

§*Centre for Condensed Matter Theory, Department of Physics, Indian Institute of Science, Bangalore 560012, India.*

E-mail: rnetz@physik.fu-berlin.de

This PDF file includes:

Sections S1 to S7

Figures S1 to S9

Table S1 to S4

References

S1. Fluctuation–dissipation theorem

The fluctuation–dissipation theorem (FDT) relates a system's equilibrium fluctuations to its response to an external perturbation.[S1] Here, we provide a derivation of the FDT (Eq. 5 in the main text) for a classical many-body system. Consider a time-dependent perturbation to a system described by the Hamiltonian

$$H(\Gamma, t) = H_0(\Gamma) - f(t)Y(\Gamma), \tag{S1}$$

where $H_0(\Gamma)$ is the unperturbed Hamiltonian of the system, dependent on phase space Γ, and $Y(\Gamma)$ is a general phase space function that the external field $f(t)$ couples to. The response of the system to $f(t)$ is quantified in terms of another phase space function $X(\Gamma)$, with its expectation value given by

$$\langle X(t)\rangle = \int d\Gamma X(\Gamma)\rho(\Gamma, t), \tag{S2}$$

where $\rho(\Gamma, t)$ is the phase space probability distribution. In the small perturbation limit, to linear order in $f(t)$, we can write the deviation in $\langle X(t)\rangle$ from its equilibrium value $\langle X\rangle_0$ as

$$\langle X(t)\rangle - \langle X\rangle_0 = \int_{-\infty}^{t} dt'\chi(t-t')f(t'), \tag{S3}$$

where the linear response function is defined by a functional derivative as

$$\chi(t-t') \equiv \left.\frac{\delta\langle X(t)\rangle}{\delta f(t')}\right|_{f=0}. \tag{S4}$$

Note that the upper limit of the integral in Eq. S3 goes up to t because of *causality*, i.e. there is no response before the perturbation. Considering a short-pulse perturbation,

$f(t) = h_0\delta(t)$, with h_0 being a constant and $\delta(t)$ the delta function, we obtain from Eq. S3

$$\langle X(t)\rangle - \langle X\rangle_0 = h_0\chi(t). \tag{S5}$$

$\rho(\Gamma, t)$ evolves according to the Liouville equation,

$$\frac{\partial\rho}{\partial t} + \{\rho, H\} = 0 \implies \frac{\partial\rho}{\partial t} = \{H_0, \rho\} - f(t)\{Y, \rho\} \equiv \mathcal{L}_0\rho - f(t)\Delta\mathcal{L}\rho, \tag{S6}$$

where $\{\}$ represents the Poisson bracket, $\mathcal{L}_0 = \{H_0, \cdot\}$, and $\Delta\mathcal{L} = \{Y, \cdot\}$. At equilibrium, the phase space probability distribution is given by $\rho_0 \propto e^{-\beta H_0}$, where $\beta = 1/k_\mathrm{B}T$, with k_B being the Boltzmann constant and T the temperature. Under the initial condition $\rho(-\infty) = \rho_0$, the solution of Eq. S6 to the first order in $f(t)$ can be written as

$$\rho(t) = \rho_0 - \int_{-\infty}^{t} dt' e^{(t-t')\mathcal{L}_0} f(t')\Delta\mathcal{L}\rho_0. \tag{S7}$$

For any general phase-space coordinate ξ, irrespective of the ensemble, we have

$$\frac{\partial\rho_0}{\partial\xi} = \frac{\partial H_0}{\partial\xi}\frac{\partial\rho_0}{\partial H_0} = -\beta\rho_0\frac{\partial H_0}{\partial\xi} \implies \{Y, \rho_0\} = -\beta\rho_0\{Y, H_0\} = -\beta\rho_0\dot{Y} \equiv \Delta\mathcal{L}\rho_0, \tag{S8}$$

where $\dot{Y}$ represents the unperturbed time evolution. Putting $\Delta\mathcal{L}\rho_0$ from Eq. S8 and $f(t) = h_0\delta(t)$ in Eq. S7 and integrating implies

$$\langle X(t)\rangle = \int d\Gamma\rho(t = 0^+)X(t, \Gamma) = \int d\Gamma\rho_0(1 + h_0\beta\dot{Y})X(t, \Gamma) = \langle X(t)\rangle_0 + h_0\beta\langle X\dot{Y}\rangle. \tag{S9}$$

Comparing Eqs. S5 and S9, we obtain the linear response of a system to an external perturbation in terms of an equilibrium property of the system, i.e., the time-correlation function in the absence of the perturbation,

$$\chi(t) = \beta\langle X(t)\dot{Y}(0)\rangle \equiv \beta C_{X\dot{Y}}, \quad t > 0. \tag{S10}$$

Using time-translation invariance, we obtain

$$C_{XY}(t) = \langle X(t)Y(0)\rangle = \langle Y(0)X(t)\rangle = \langle Y(-t)X(0)\rangle = C_{YX}(-t). \tag{S11}$$

The time derivative of the correlation function $C_{XY}(t)$ can be written as

$$\frac{d}{dt}C_{XY}(t) = \frac{d}{dt}\int d\Gamma \rho_0(\Gamma)X(\Gamma,t)Y(\Gamma) = \int d\Gamma \rho_0(\Gamma)\frac{dX}{dt}Y = C_{\dot{X}Y}(t). \tag{S12}$$

Combining Eqs. S10–S12, we obtain

$$\begin{aligned}\chi(t) &= -\beta\frac{d}{dt}C_{XY}(t), \quad t > 0\\ &= 0, \quad t < 0,\end{aligned}$$

or in the compact form as in Eq. 5 in the main text

$$\boxed{\chi(t) = -\beta\theta(t)\frac{d}{dt}C_{XY}(t),} \tag{S13}$$

where $\theta(t)$ is the Heaviside step function.

S2. Order parameters for characterizing the coiled-coil (CC) structure

The *fraction of native contacts*, Q, is defined as[S2]

$$Q(t) = \frac{1}{N_c}\sum_{(i,j)}\frac{1}{1+\exp\left(\lambda[r_{ij}(t) - \nu r^0_{ij}]\right)}, \tag{S14}$$

where the sum runs over the N_c pairs of native atomic contacts (i,j), $r_{ij}(t)$ and r^0_{ij} are the distances between atoms i and j at time t and in the native structure of the protein, $\lambda = 5$ Å^{-1} is a smoothing parameter, and $\nu = 1.8$ is an offset parameter. The list of native contact

pairs (i, j) is constructed by considering all pairs of heavy atoms belonging to residues L and M such that $|L - M| > 3$ and $r_{ij}^0 < 4.5$ Å.

Root-mean-square deviation, RMSD, in distances is defined as

$$RMSD(t) = \sqrt{\frac{1}{N}\sum_{i=1}^{N} |r_i(t) - r_i^0|^2}, \tag{S15}$$

where N is the total number of heavy atoms of the CC, $r_i(t)$ is the position of the ith atom at time t, and r_i^0 is the position of the same atom in the CC native structure from the PDB database (ID: 4GCZ).

Secondary structure, SS, contents of the CC are calculated by the STRIDE algorithm[S3] implemented in the VMD software.[S4] The fraction of SS content is defined as the ratio of the number of residues of the protein involved in α-helical SS formation at time t to that in the CC native structure from the PDB database (ID: 4GCZ).

S3. Force transmission through a viscous bead–spring model.

To illustrate force transmission through a model signal transmitter, we consider a simple viscoelastic model: two beads with different mobilities μ_{s} (for the sensor) and μ_{e} (for the effector), connected by a harmonic spring of stiffness k, similar to Figure 1B in the main text.[S5] Under the application of oscillating forces with amplitudes $f_{\text{s}}(\omega)$ and $f_{\text{e}}(\omega)$ to the sensor and effector, respectively, the Fourier-transformed sensor velocity is given by $-i\omega\tilde{x}_{\text{s}}(\omega) = \mu_{\text{s}}(f_{\text{s}}(\omega) + k[\tilde{x}_{\text{e}}(\omega) - \tilde{x}_{\text{s}}(\omega)])$, whereas the effector velocity is given by $-i\omega\tilde{x}_{\text{e}}(\omega) = \mu_{\text{e}}(f_{\text{e}}(\omega) + k[\tilde{x}_{\text{s}}(\omega) - \tilde{x}_{\text{e}}(\omega)])$. Using the definitions of the linear response functions from Eq. 1 in the main text, we obtain

$$\tilde{J}^{\text{s}}_{\text{self}} = \frac{\mu_{\text{s}}(\omega + i\mu_{\text{e}}k)}{\omega(\mu k - i\omega)}, \quad \tilde{J}^{\text{e}}_{\text{self}} = \frac{\mu_{\text{e}}(\omega + i\mu_{\text{s}}k)}{\omega(\mu k - i\omega)}, \quad \tilde{J}_{\text{cross}} = \frac{i\mu_{\text{s}}\mu_{\text{e}}k}{\omega(\mu k - i\omega)}, \tag{S16}$$

where $\mu = \mu_\mathrm{s} + \mu_\mathrm{e}$. The force transmission from the sensor to the effector side is determined according to Eq. 2 in the main text as

$$\tilde{T}_\mathrm{F}^{\mathrm{s}\to\mathrm{e}}(\omega) = \frac{\tilde{J}_\mathrm{cross}(\omega)}{\tilde{J}^\mathrm{e}_\mathrm{self}(\omega)} = \frac{\mu_\mathrm{s} k}{(\mu_\mathrm{s} k - i\omega)}. \tag{S17}$$

Note that $\tilde{T}_\mathrm{F}^{\mathrm{s}\to\mathrm{e}}$ is independent of the effector side bead mobility, and at zero frequency, which corresponds to the long-time limit, the force transmission is given by $\tilde{T}_\mathrm{F}^{\mathrm{s}\to\mathrm{e}} = 1$. The derived force transmit function has the same form as the Debye function. For more realistic force transducers, such as proteins, $\tilde{T}_\mathrm{F}$ can be expressed as a sum of Debye functions that reflect the normal modes of a protein.

S4. Fitting procedure for the analytical representation of a response function

We consider multi-exponential functions for fitting the response function $J(t)$ according to

$$J(t) = \theta(t) \sum_{k=1}^{N} c_k e^{-t/\tau_k} = \sum_{k=1}^{N} g_k(t), \tag{S18}$$

where $\theta(t)$ is the Heaviside step function given by

$$\theta(t) = \begin{cases} 1, & t \geq 0 \\ 0, & t < 0. \end{cases}$$

The Fourier transform of a single-sided exponential is the Debye function given by

$$\tilde{g}_k(\omega) = \frac{c_k}{\tau_k^{-1} + i\omega} = \frac{c_k \tau_k}{1 + \tau_k^2 \omega^2} - i \frac{c_k \tau_k^2 \omega}{1 + \tau_k^2 \omega^2}. \tag{S19}$$

The real part of the Fourier-transformed fit function is given by

$$\Re\ [\tilde{J}(\omega)] = \Re\big[\sum_{k=1}^{N} \tilde{g}_k(\omega)\big] = \sum_{k=1}^{N} \frac{c_k \tau_k}{1+\tau_k^2\omega^2}, \tag{S20}$$

and the imaginary part is given by

$$\Im\ [\tilde{J}(\omega)] = \Im\big[\sum_{k=1}^{N} \tilde{g}_k(\omega)\big] = -\sum_{k=1}^{N} \frac{c_k \tau_k^2 \omega}{1+\tau_k^2\omega^2}. \tag{S21}$$

Eqs. S20,S21 are used to simultaneously fit the real and imaginary parts of the Fourier-transformed response functions. Logarithmically-spaced frequency data are taken for the fitting. The time constants, τ_k, in Eq. S18 are restricted to positive values only. The number of Debye functions, N, used for fitting the self and cross-response functions for the different systems are given in Table S1. Such fits for the twist, shift, and splay modes of the AcNH-CC-$CONH_2$ system are shown in Figure 2C,D (in the main text) and Figure S3C,D. Importantly, we find that the parameters c_k and τ_k obtained from the frequency-domain fitting accurately reproduce the time-domain response functions from simulations, validating the robustness of our fitting procedure (see Figure 2B in the main text and Figure S3B).

Table S1: Number of Debye relaxation functions, N, used for fitting self and cross-response functions for the different isolated CCs.

System	$N(\tilde{J}_{\text{self}})$	$N(\tilde{J}_{\text{cross}})$
AcNH-CC-$CONH_2$	10	10
NH_3^+-CC-$CONH_2$	7	10
AcNH-CC[Q133L]-$CONH_2$	7	10
AcNH-CC[R135L]-$CONH_2$	7	10

S5. Time-domain transmit function from the analytical form of $\tilde{T}_{\mathrm{F}}(\omega)$ via partial fraction decomposition

The time-domain force transmit function, $T_{\mathrm{F}}(t)$, corresponds to the system response to a δ-function input pulse and is given by the inverse Fourier transform of the Fourier-domain transmit function, $\tilde{T}_{\mathrm{F}}(\omega)$, according to $T_{\mathrm{F}}(t) = \frac{1}{2\pi}\int_{-\infty}^{+\infty} \tilde{T}_{\mathrm{F}}(\omega)e^{i\omega t}d\omega$. Instead of using a numerical discrete Fourier transform, we obtain $T_{\mathrm{F}}(t)$ from $\tilde{T}_{\mathrm{F}}(\omega)$ analytically as follows. Since all response functions $\tilde{J}(\omega)$ are expressed as sums of Debye functions (see Section S4), the Fourier-transformed force transmit function is given by

$$\tilde{T}_{\mathrm{F}}(\omega) = \frac{\tilde{J}_{\mathrm{cross}}(\omega)}{\tilde{J}_{\mathrm{self}}(\omega)} = \sum_{j=1}^{M} \frac{a_j}{\alpha_j + i\omega} \Bigg/ \sum_{k=1}^{N} \frac{b_k}{\beta_k + i\omega}, \tag{S22}$$

where a_j, α_j, b_k, and β_k are the fitting parameters. The above expression can be rewritten as a sum of Debye functions using partial fraction decomposition as

$$\tilde{T}_{\mathrm{F}}(\omega) = c_0 + \sum_{l=1}^{Q} \frac{c_l}{\gamma_l + i\omega}, \tag{S23}$$

where c_0, c_l, and γ_l are uniquely determined parameters, provided for the isolated CC's deformation modes in Tables S2–S4. Based on Eq. S23, the analytical inverse Fourier transform is obtained as

$$T_{\mathrm{F}}(t) = c_0\delta(t) + \theta(t)\sum_{l=1}^{Q} c_l e^{-\gamma_l t}, \tag{S24}$$

where $\theta(t)$ is the Heaviside step function.

Once the transmit function $T_{\mathrm{F}}(t)$ is known, the transmitted force $F_e(t)$ at the effector site follows from the input force $F_s(t)$ at the sensor site by convolution (see Eq. 6 in the

main text). For a step input force signal $F_s(t) = F_0\theta(t)$, we obtain

$$F_{\mathrm{e}}^{\mathrm{stp}}(t) = F_0 \int_0^{\infty} T_{\mathrm{F}}(t')\theta(t-t')dt' = F_0 \int_0^{t} T_{\mathrm{F}}(t')dt'$$

$$\implies F_{\mathrm{e}}^{\mathrm{stp}}(t)/F_0 = c_0\theta(t) + \sum_{l=1}^{Q} \frac{c_l}{\gamma_l}(1-e^{-\gamma_l t}). \tag{S25}$$

Similarly, the rectangular pulse response is given by

$$F_{\mathrm{e}}^{\mathrm{rec}}(t) = F_0 \int_0^{\infty} T_{\mathrm{F}}(t')\Pi(t-t')dt',$$

where $\Pi(t)$ is a rectangular pulse of width τ defined as

$$\Pi(t) = \begin{cases} 0 & t < 0 \\ 1 & 0 \leq t \leq \tau \\ 0 & t > \tau. \end{cases}$$

The above convolution integral is evaluated to be

$$F_{\mathrm{e}}^{\mathrm{rec}}(t)/F_0 = \begin{cases} \int_0^t T_{\mathrm{F}}(t')dt' & 0 \leq t \leq \tau \\ \int_{t-\tau}^t T_{\mathrm{F}}(t')dt' & t > \tau \end{cases} = \begin{cases} c_0 + \sum_{l=1}^{Q} \frac{c_l}{\gamma_l}(1-e^{-\gamma_l t}) & 0 \leq t \leq \tau \\ \sum_{l=1}^{Q} \frac{c_l}{\gamma_l} e^{-\gamma_l t}(e^{\gamma_l \tau}-1) & t > \tau. \end{cases} \tag{S26}$$

Table S2: Parameters obtained from the partial fraction decomposition of the the shift mode sensor-to-effector ($s \to e$) and effector-to-sensor ($e \to s$) transmit functions $\tilde{T}_{\mathrm{F}}$ (see Eq. S23) for the isolated coiled coil, AcNH-CC-CONH$_2$.

shift($s \to e$) [a]		shift($e \to s$) [b]	
c_l [1/ps]	$1/\gamma_l$ [ps]	c_l [1/ps]	$1/\gamma_l$ [ps]
-0.4437022732226056	2964.2801620821574	-0.42549293303447067	2834.3921053966496
0.9948280388059464	2834.3921053966496	1.0573042003363176	2730.0419119656326
-0.9376226344275997	2584.4475228674655	-0.8401311017782265	2584.4475228674655
-1.4956479460236798e-13	2496.6504153197657	0.32477980434227144	2231.976899522938
0.6465325249580955	2231.976899522938	-0.1725684174483331	1929.4343085934504
-0.39495343961443513	1929.4343085934504	0.056116658093106654	1762.0045848330863
0.13496432917221798	1762.0045848330863	-1.9996719614980007e-05	258.7949724201136
-7.169643704009382e-16	1175.7680115630044	-5.11818479443269e-09	70.60332578698359
5.6919909659780554e-06	318.29284088841183	-3.213494694142265e-09	65.44728935751215
-0.0006070332503173986	82.82348528634269	-0.024261832534937633	32.36075345417574
-0.352147736659321	24.68444274170711	0.6858694833370592	24.68444274170711
0.8523978190837392	23.622796349390665	-0.9814084884148848	23.622796349390665
-0.6569941231470044	21.966172839963004	0.31992542921479267	21.966172839963004
0.1603756614719739	19.03133443695339	0.0011222569166474787	8.670408821419079
1.0527118977280836e-08	4.928051928768867	0.007623485062426466	4.135338290033818
0.009207561711270795	4.555178770900826	-0.0905874748082455	2.3247294797363756
-0.15830468110894455	2.3247294797363756	0.08438328935784299	2.2167939787900375
0.16032360930455924	2.2167939787900375	0.055228926848408855	0.6645701468457365
0.05255462689631596	0.5734403320679259	0.00012029376650133034	1.411281442258735e-07

[a] c_0 = 1.7550490830635147e-38; [b] c_0 = 1.0442674420574836e-11.

Table S3: Parameters obtained from the partial fraction decomposition of the the splay mode sensor-to-effector ($s \to e$) and effector-to-sensor ($e \to s$) transmit functions $\tilde{T}_{\mathrm{F}}$ (see Eq. S23) for the isolated coiled coil, AcNH-CC-CONH$_2$.

splay($s \to e$) [a]		splay($e \to s$) [b]	
c_l [1/ps]	$1/\gamma_l$ [ps]	c_l [1/ps]	$1/\gamma_l$ [ps]
-4.600345406149606e-05	5031.915219531668	4.425700819280955e-17	5402.29234230296
0.06350375727464268	2166.3865113878214	-0.0012734866692581184	3061.5862167163614
-0.13745056471970557	2022.5767169493288	0.08116585587839227	2166.3865113878214
0.08001949002218163	1850.7574308358824	-0.15814167922561384	2022.5767169493288
-0.04146659328209217	802.4724465718533	0.08310559175334685	1850.7574308358824
-0.08668203075637121	712.5786068408191	-0.07402607970912835	802.4724465718533
0.5652066909046669	622.0508483669215	0.12691572017208883	712.5786068408191
-0.6854859856487758	571.1818018875739	-0.07456253369759723	622.0508483669215
0.2424863992933528	526.1718910871631	0.01630638824873847	571.1818018875739
0.0004728870424610676	78.04044172050372	4.678248872029564e-05	523.9312095029796
1.5355828409782762e-13	36.645691126101774	2.844259391201826e-08	495.51895539300847
-0.659524648399697	34.464517243411535	7.929982363442378e-08	351.56085820762223
1.2630435495040258	33.80229117609624	0.0005663597259893821	225.31265511867352
-0.6024961605867062	33.1463514678304	0.00023865556546811412	60.86148723579737
-1.798379662891735e-13	17.216568554432694	1.4966183844997598	34.464517243411535
-0.0014499354526273378	15.88890318360671	-3.229814418967065	33.80229117609624
-0.00038641114378387686	3.913767238215506	1.7438671898742575	33.1463514678304
6.6774420922206556e-15	1.6949841492762274	-0.010790834707430252	20.272158186288852
-0.0009531402085832807	0.6529268578662905	-0.0014170147750273143	1.8802016262442127

[a] c_0 = -1.1059706892655322e-38; [b] c_0 = -0.00012605785091458765.

Table S4: Parameters obtained from the partial fraction decomposition of the the twist mode sensor-to-effector ($s \to e$) and effector-to-sensor ($e \to s$) transmit functions $\tilde{T}_{\mathrm{F}}$ (see Eq. S23) for the isolated coiled coil, AcNH-CC-$CONH_2$.

twist($s \to e$) [a]		twist($e \to s$) [b]	
c_l [1/ps]	$1/\gamma_l$ [ps]	c_l [1/ps]	$1/\gamma_l$ [ps]
-0.0029984391713186553	2896.2854031967486	-0.0032648755534184027	2896.2854031967486
0.002769072910820333	2692.0832821655376	0.0041140753108451665	2692.0832821655376
0.007277089620122361	2417.2571393249927	8.699485335016213e-09	2531.937620394691
-0.008009015362338596	2232.2744191711968	0.003920327736315615	2417.2571393249927
0.0010465339071473595	1427.590345114574	-0.005355074770296874	2232.2744191711968
1.0101124456175489e-08	759.9772834708515	-5.729145869010972e-15	1299.9699044629415
1.2795041147965367e-07	501.5347384235006	0.0006997294972538176	1192.6097437620776
0.00017974149434444968	402.7970956029895	0.050544998363096186	281.9934811820565
-0.02444799094303891	281.9934811820565	-0.057334689949221716	278.89380955584227
0.024311776516237133	278.89380955584227	0.007090469420105817	241.73825811688394
4.5314758437521927e-07	157.15570566760746	1.2278619526293554e-06	91.63256355593118
0.0003427081270030233	100.01238234638262	1.647925405475273e-05	87.11672610934001
-0.050253247025102095	24.737354206692558	-2.796772975960781	24.769187191104045
0.05557068839794185	23.190461670622984	2.802291789864151	24.737354206692558
-0.024365889931671894	4.863254792531972	-0.021115537391879657	5.271573928096085
0.06297119096634829	1.7582427570779058	0.061918167379104855	1.7582427570779058
0.025507332873022925	0.5691872744867393	0.026510721654032288	0.5702587879017043
-3252.168559922061	0.10014024508066352	-3333.512734277682	0.10014024508066352
3258.9486198323925	0.10000002266655493	3340.466527622838	0.10000002266655493

[a] c_0 = 8.629872517163786e-16; [b] c_0 = 3.295611601470914e-36.

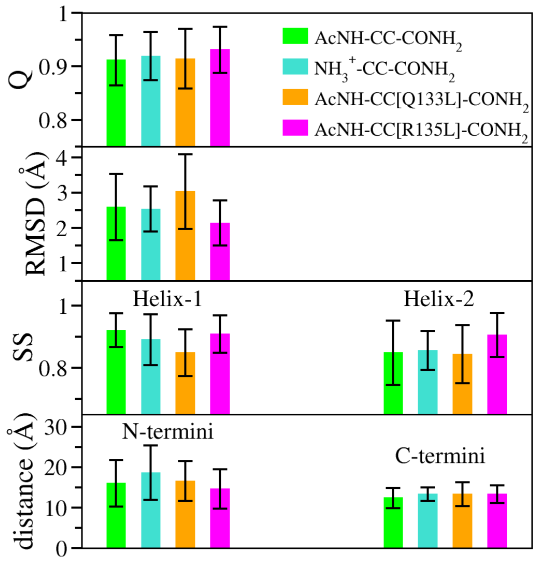

Figure S1: Average values of different structural order parameters for the four simulated coiled-coil systems: AcNH-CC-CONH$_2$, NH$_3^+$-CC-CONH$_2$, AcNH-CC[Q133L]-CONH$_2$, and AcNH-CC[R135L]-CONH$_2$. See Section S2 for the definitions of the order parameters. The results demonstrate that all four systems are stable over the total simulation time of 20 μs each.

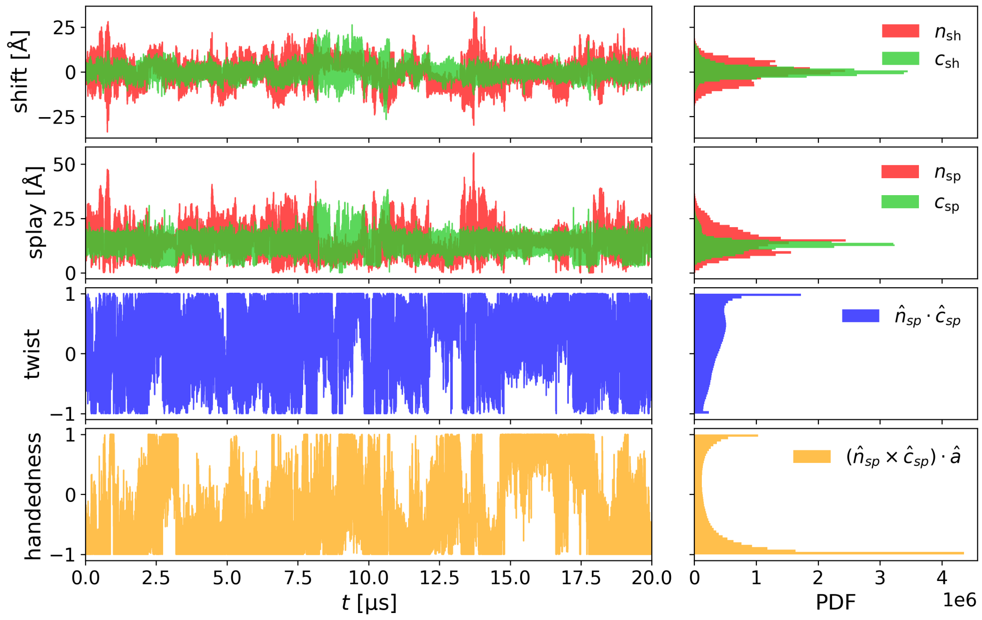

Figure S2: Time series and corresponding probability distribution functions for the shift, the splay, the cosine of the twist angle between N and C termini, and the handedness of the rotation of the coiled coil along its long-axis $\vec{a}$, defined as $(\hat{n}_{\rm sp} \times \hat{c}_{\rm sp}) \cdot \hat{a}$. See Figure 1F,G and the main text for the definitions of shift, splay, and the twist angle. Results are shown for the wild-type CC with capped neutral termini (AcNH-CC-CONH$_2$).

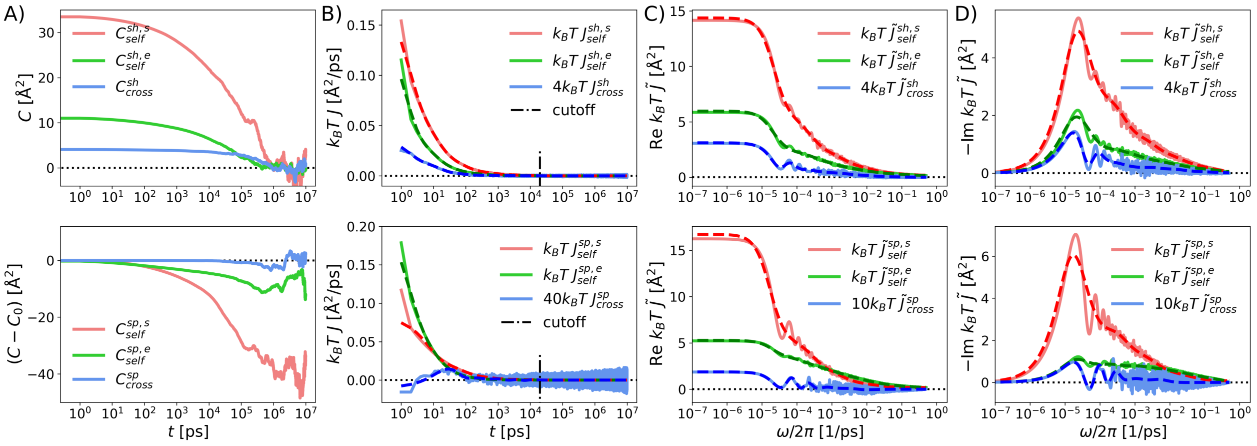

Figure S3: Correlation and response functions for the shift (top) and splay modes (bottom) of the wild-type CC with capped neutral termini (AcNH-CC-CONH$_2$). A) Self and cross-correlation functions C_{self} and C_{cross}. For the splay mode, we subtract $C_0 = C(t = 0)$ in order to be able to compare all correlation functions in one plot. B) Response functions $J(t)$, obtained from the numerical time derivatives of the correlation functions, are shown as solid lines. The vertical dash-dotted lines represent the cutoff beyond which the response functions are set to zero to prevent noise artifacts when calculating Fourier transforms. C) Real and D) imaginary parts of $\tilde{J}(\omega)$ obtained from discrete fast-Fourier transformation of the time-domain response function $J(t)$, shown as solid lines. Dashed lines represent simultaneous fits to the logarithmically-spaced real and imaginary parts of $\tilde{J}(\omega)$ by a sum of 10 Debye functions (for details, see Section S4). Dashed lines in panel B represent the inverse Fourier transform of the fits in C and D. Note that the cross-response functions in B, C, and D are multiplied by different scaling factors for better visualization.

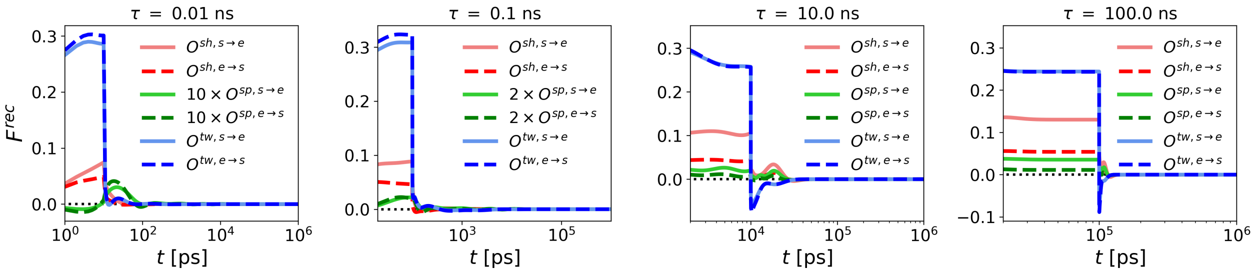

Figure S4: Transmitted force profiles for a rectangular pulse force input for shift, splay, and twist modes of the wild-type CC with capped neutral termini (AcNH-CC-CONH$_2$) for different input pulse widths τ given on the top of each plot. Note that for the splay mode, transmitted force profiles are multiplied by a different scaling factor in each plot for better visualization.

S6. Simulation results of the full histidine kinase protein YF1

We have used the 2 μs-long, explicit solvent all-atom MD simulation trajectory of the whole histidine kinase protein (see Methods in the main text for simulation details, and the simulation box in Figure S5), to check how the presence of sensor and effector modules affects the dynamics of the CC linker and the signal transfer efficacy of its different modes. We expect that the 2 μs MD simulation is adequate to capture the relevant dynamics of the whole protein, as the self and cross response functions for each transmission mode decay to zero within 100 ns (Figure S6). In terms of the force transmission efficiencies of the different modes, we find qualitatively similar simulation results for the isolated CC system shown in Figure 3 in the main text and the whole protein shown in Figure S7 (see the low-frequency plateau values of the real part of force transmit functions, Re $\tilde{T}_{\mathrm{F}}$). The force transmission through the twist mode is highest, followed by that through the shift and splay modes. The low-frequency plateau value of Re $\tilde{T}_{\mathrm{F}}$ for each mode, however, is larger for the whole protein simulation, compared to the isolated CC simulation. This is due to the relative modulation of self and cross response functions, while the low-frequency plateau values of self and cross response functions obtained from the whole protein simulation (Figure S6C) are significantly smaller compared with that obtained from the simulation of the isolated CC system, AcNH-CC-CONH$_2$ (Figure 2C in the main text and Figure S3C). We conclude that the reduced CC terminal fluctuations in the full construct in fact increase the signal transmission efficiency compared to the isolated CC scenario.

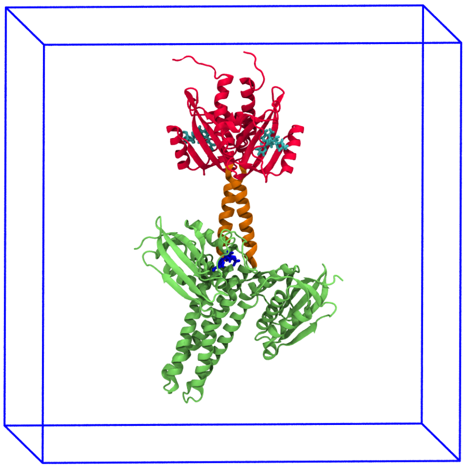

Figure S5: Simulation unit cell containing the homodimeric protein histidine kinase YF1 with two FMN cofactors (cyan) and one ADP (blue) bound to its sensor (red) and effector (green) modules, respectively. The CC linker is shown in orange. Water molecules and ions are not shown.

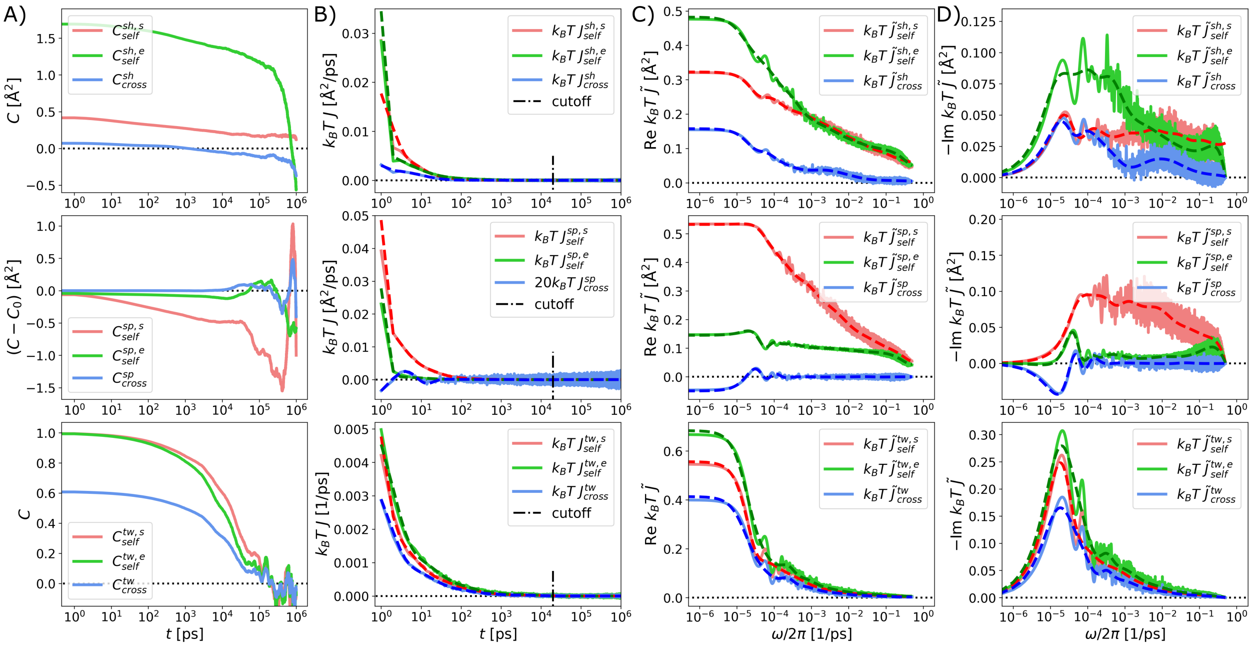

Figure S6: Correlation and response functions obtained from the simulation of the whole histidine kinase YF1 for its coiled-coils' shift (sh), splay (sp), and twist (tw) modes, shown in top-to-bottom rows. A) Self and cross-correlation functions C_{self} and C_{cross}. For the splay mode, we subtract $C_0 = C(t = 0)$ to be able to compare all correlation functions in one plot. B) Response functions $J(t)$, obtained from the numerical time derivatives of the correlation functions, are shown as solid lines. The vertical dash-dotted lines represent the cutoff beyond which the response functions are set to zero to prevent noise artifacts when calculating Fourier transforms. C) Real and D) imaginary parts of $\tilde{J}(\omega)$ obtained from discrete fast-Fourier transformation of the time-domain response function $J(t)$, shown as solid lines. Dashed lines represent simultaneous fits to the logarithmically-spaced real and imaginary parts of $\tilde{J}(\omega)$ by a sum of 10 Debye functions (the same number as for the isolated, wild-type CC system), for further details, see Section S4. Dashed lines in panel B represent the inverse Fourier transform of the fits in C and D. Note that $J^{\text{sp}}_{\text{cross}}$ in panel B is multiplied by a factor of 20 for better visualization.

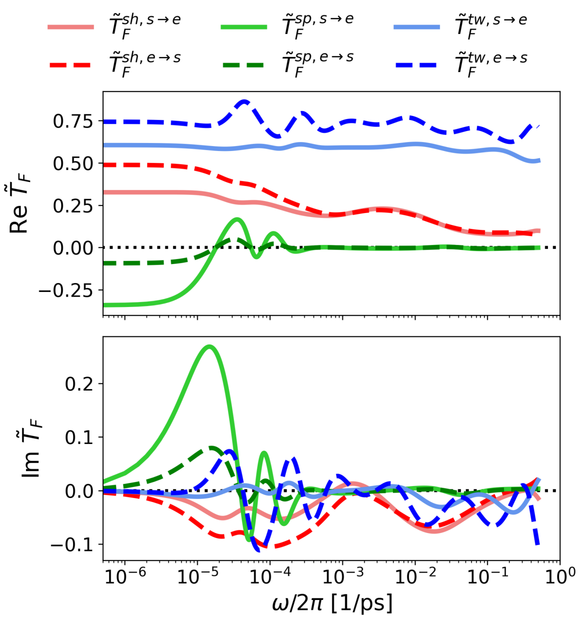

Figure S7: Force transmit functions, $\tilde{T}_{\mathrm{F}}$, from the simulation of the whole histidine kinase YF1. (top) Real and (bottom) imaginary part of $\tilde{T}_{\mathrm{F}}$, obtained using analytical representations for the self and cross-response functions (details in Section S4) according to Eq. 2 in the main text, for the CC shift (sh), splay (sp), and twist (tw) modes. The sensor-to-effector (s $\rightarrow$ e) and effector-to-sensor (e $\rightarrow$ s) transmit functions are shown as solid and dashed lines, respectively.

S7. Force field effects on signal transfer through the isolated wild-type CC

To check the effect of force field parameters, we have also performed simulations of the wild-type CC capped with neutral termini (AcNH-CC-$CONH_2$), using Amber99SB-ILDN protein[S6,S7] in combination with TIP3P water,[S8] as well as using DES-Amber protein[S9,S10] in combination with the recommended TIP4P-D water.[S11] The Amber99SB-ILDN and DES-Amber simulations are of durations 3.0 μs and 2.6 μs, respectively. The simulation set-up and all simulation parameters are the same as those provided in the main text for the isolated CC simulations using CHARMM36m,[S12] except a cutoff distance of 1.0 nm used for the real-space part of the particle mesh Ewald electrostatic computation and for the computation of the van der Waals interactions where the Lennard-Jones potential is shifted by a constant such that it is zero at the cutoff. Furthermore, the long-range dispersion corrections for energy and pressure have been applied, in contrast to the CHARMM36m simulations.

To check the stability of secondary and tertiary structures of the isolated CC in simulations using the different force fields, the average values of the fraction of native contacts Q between the two α-helices, the root-mean-square deviation (RMSD) of distances between the native structure from the PDB database (ID: 4GCZ) and the simulated CC structures, and the secondary structure (SS) content—all defined in Section S2—are provided in Figure S8A. Compared to the CHARMM36m and DES-Amber results, we find the CC structure is less stable in the Amber99SB-ILDN simulation as becomes clear from the higher RMSD value and lower Q and SS values. The effector side C-termini distance is also comparatively larger in the Amber99SB-ILDN simulation. Therefore, one has to carefully choose force fields known to reproduce both folded and disordered ensembles equally well, especially for simulations of protein fragments. To check how force field parameters affect signal transfer through the CC, we compare the time-domain force transmit functions $T_F^{sh}(t)$ for the shift mode and find comparable results for CHARMM36m and DES-Amber (see Figure S8B). These results show that the signal transfer dynamics extracted from the CHARMM36m and

DES-Amber simulations, which both reproduce the native CC structure very well, are rather similar, which demonstrates the robustness of our approach. The Amber99SB-ILDN simulation yields rather different signal transfer dynamics, which is not surprising since it fails to reproduce the native CC structure. In the future it would be interesting to study the signal transfer dynamics of the whole histidine kinase protein and of different proteins from extensive MD simulations with different force fields.

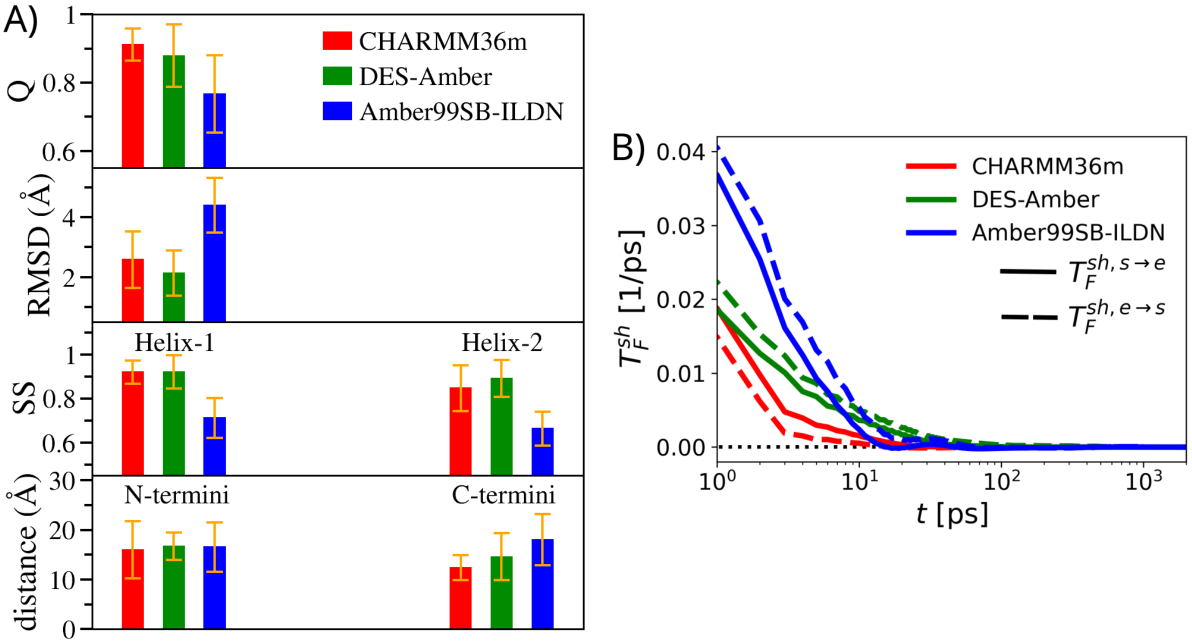

Figure S8: Comparison of results for the isolated coiled coil, AcNH-CC-$CONH_2$, from simulations performed using three different protein force fields: CHARMM36m, DES-Amber, and Amber99SB-ILDN. A) Average values of different structural order parameters defined in Section S2, to quantify the conformational stability of the coiled coil. B) Time-domain force transmit functions T_F^{sh} for the shift mode from the sensor-to-effector ($s \to e$) and effector-to-sesor ($e \to s$) sides shown as solid and broken lines, respectively.

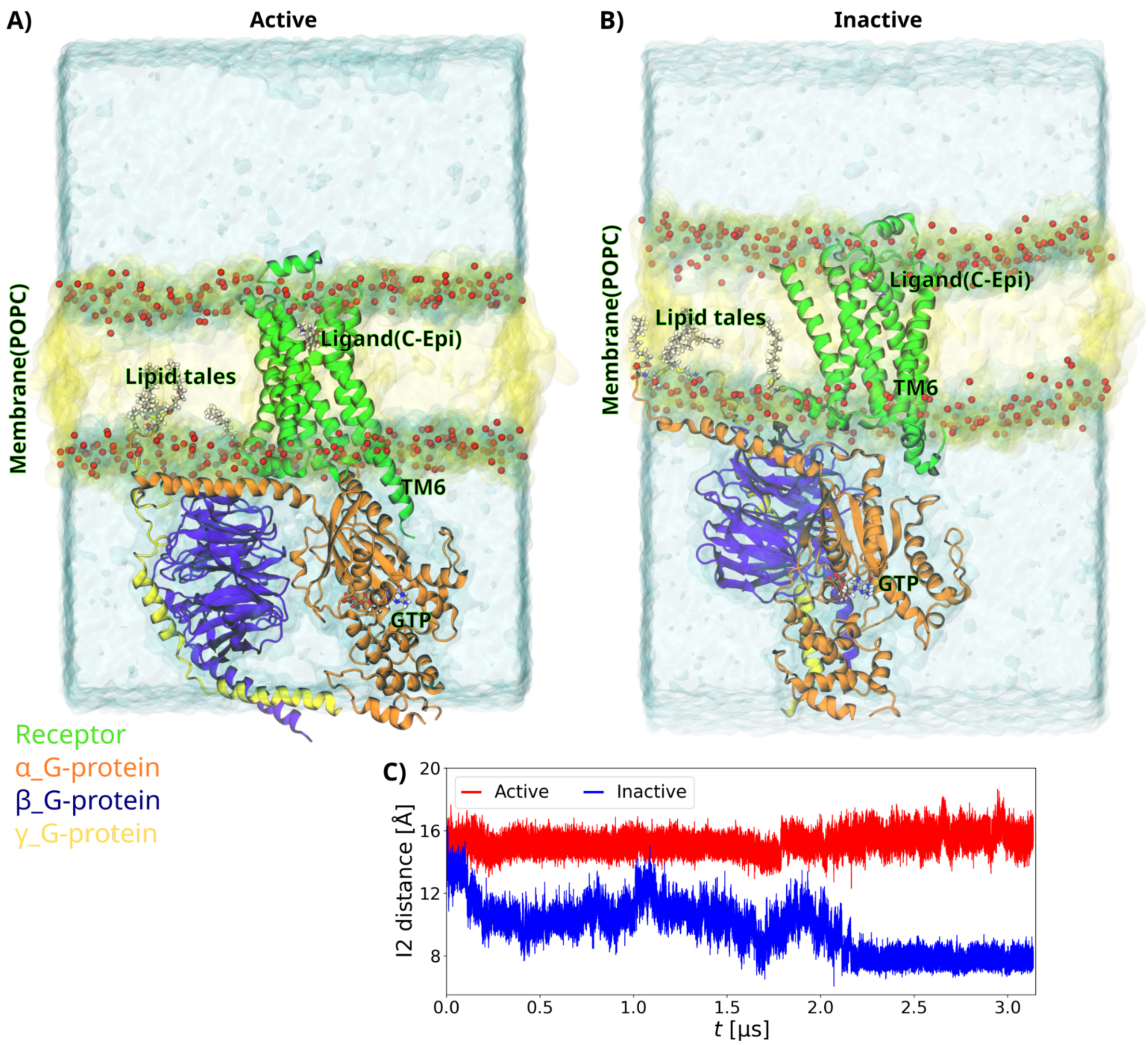

Figure S9: Snapshots of simulated systems of the GPCR β_2-adrenergic receptor protein in A) active and B) inactive states, taken at time $t = 3$ μs. The intracellular part of the transmembrane helix 6 (TM6) remains in the open or close conformation in the active or inactive state, respectively, as monitored by C) the timeseries of I2 (distance between the backbone atoms of R131 and L272 as depicted in Figure 6 in the main text). For further details, see the main text.

References

(S1) Kubo, R. The fluctuation-dissipation theorem. *Rep. Prog. Phys.* **1966**, *29*, 255.

(S2) Best, R. B.; Hummer, G.; Eaton, W. A. Native contacts determine protein folding mechanisms in atomistic simulations. *Proc. Natl. Acad. Sci. USA* **2013**, *110*, 17874–17879.

(S3) Frishman, D.; Argos, P. Knowledge-based protein secondary structure assignment. *Proteins: Struct., Funct., Bioinf.* **1995**, *23*, 566–579.

(S4) Humphrey, W.; Dalke, A.; Schulten, K. VMD: visual molecular dynamics. *J. Mol. Graph.* **1996**, *14*, 33–38.

(S5) Hinczewski, M.; von Hansen, Y.; Netz, R. R. Deconvolution of dynamic mechanical networks. *Proc. Natl. Acad. Sci. USA* **2010**, *107*, 21493–21498.

(S6) Hornak, V.; Abel, R.; Okur, A.; Strockbine, B.; Roitberg, A.; Simmerling, C. Comparison of multiple Amber force fields and development of improved protein backbone parameters. *Proteins: Struct., Funct., and Bioinf.* **2006**, *65*, 712–725.

(S7) Lindorff-Larsen, K.; Piana, S.; Palmo, K.; Maragakis, P.; Klepeis, J. L.; Dror, R. O.; Shaw, D. E. Improved side-chain torsion potentials for the Amber ff99SB protein force field. *Proteins: Struct., Funct., and Bioinf.* **2010**, *78*, 1950–1958.

(S8) Jorgensen, W. L.; Chandrasekhar, J.; Madura, J. D.; Impey, R. W.; Klein, M. L. Comparison of simple potential functions for simulating liquid water. *J. Chem. Phys.* **1983**, *79*, 926–935.

(S9) Piana, S.; Robustelli, P.; Tan, D.; Chen, S.; Shaw, D. E. Development of a force field for the simulation of single-chain proteins and protein–protein complexes. *J. Chem. Theory Comput.* **2020**, *16*, 2494–2507.

(S10) Robustelli, P.; Piana, S.; Shaw, D. E. Developing a molecular dynamics force field for both folded and disordered protein states. *Proc. Natl. Acad. Sci. USA* **2018**, *115*, E4758–E4766.

(S11) Piana, S.; Donchev, A. G.; Robustelli, P.; Shaw, D. E. Water dispersion interactions strongly influence simulated structural properties of disordered protein states. *J. Phys. Chem. B* **2015**, *119*, 5113–5123.

(S12) Huang, J.; Rauscher, S.; Nawrocki, G.; Ran, T.; Feig, M.; De Groot, B. L.; Grubmüller, H.; MacKerell, A. D. CHARMM36m: an improved force field for folded and intrinsically disordered proteins. *Nat. Methods* **2017**, *14*, 71–73.
